# Prescribing cascades potentially associated with harms before and after transition to long-term care facilities

**DOI:** 10.1093/ageing/afag166

**Published:** 2026-06-18

**Authors:** Craig Hansen, Lisa Kalisch Ellett, Tracy Air, Maria C Inacio, Gillian Elizabeth Caughey

**Affiliations:** Registry of Senior Australians Research Centre, College of Nursing and Health Sciences, Flinders University, Bedford Park, South Australia, Australia; Registry of Senior Australians Research Centre, South Australian Health and Medical Research Institute Limited, Adelaide, South Australia, Australia; School of Pharmacy and Biomedical Science, College of Health, Adelaide University, Australia; Registry of Senior Australians Research Centre, College of Nursing and Health Sciences, Flinders University, Bedford Park, South Australia, Australia; Registry of Senior Australians Research Centre, South Australian Health and Medical Research Institute Limited, Adelaide, South Australia, Australia; Registry of Senior Australians Research Centre, College of Nursing and Health Sciences, Flinders University, Bedford Park, South Australia, Australia; Registry of Senior Australians Research Centre, South Australian Health and Medical Research Institute Limited, Adelaide, South Australia, Australia; Registry of Senior Australians Research Centre, College of Nursing and Health Sciences, Flinders University, Bedford Park, South Australia, Australia; Registry of Senior Australians Research Centre, South Australian Health and Medical Research Institute Limited, Adelaide, South Australia, Australia

**Keywords:** medications, prescribing cascades, aged care, dementia, older people

## Abstract

**Background:**

Transition to long-term care facilities (LTCFs) is a critical period associated with increases in medication errors and adverse events, yet our knowledge of prescribing cascades in the older population during this period is limited. The objective was to identify prescribing cascades potentially associated with harm in LTCF residents before and after care entry.

**Methods:**

A retrospective population-based cohort study of 167 850 individuals aged 65 years or older who entered 2771 LTCFs nationally between January 2018 and June 2020 was conducted. Prescription sequence symmetry analysis was used to examine 142 prescribing cascades with a 12-month exposure window between the index and marker medicines.

**Results:**

Among 167 850 residents, 16.7% (*n* = 28 018) had at least one statistically significant prescribing cascade before transition to LTCF and 25.1% (*n* = 42 101) after LTCF, with little difference in prevalence when stratified by dementia status. Before LTCF entry, initiation of statins was associated with initiation of multiple medications [e.g. antipsychotics adjusted sequence ratio (aSR) 2.97, 95% confidence interval (CI) 2.58–3.41], antidepressants (aSR 1.60, 95% CI 1.45–1.76) and benzodiazepines were associated with initiation of antipsychotics (aSR 1.43, 95% CI 1.35–1.52). After LTCF entry, initiation of statins was associated with multiple medications (e.g. antipsychotics aSR 2.93, 95% CI: 2.59–3.3 and antidepressants aSR 2.29, 95% CI: 2.05–2.56). Initiation of benzodiazepines was associated with antipsychotics (aSR 1.53, 95% CI: 1.47–1.59). Six cascades prior to LTCF entry and 13 after were unique to residents living with dementia with many associated with insomnia/sedation.

**Conclusion:**

This national population-based study identified prescribing patterns consistent with potential prescribing cascades during a vulnerable care transition.

## Key points

- Of 167 850 LTCF residents, 16.7% had at least one prescribing cascade before transition to LTCF and 25.1% after.
- Many prescribing cascades involved high-risk medications such as antipsychotics, benzodiazepines and opioids.
- Routine medication review and deprescribing initiatives targeting known prescribing cascades will improve medication safety.

## Introduction

Prescribing cascades occur when an adverse drug reaction is interpreted as a new symptom or medical condition, leading to the prescription of a new medication [1]. While some prescribing cascades may be considered appropriate (e.g. concordant with guideline recommendations), many are considered to be inappropriate (e.g. an adverse drug reaction is misinterpreted as a new condition) [2]. Prescribing cascades increase medication regimen complexity and risk of further adverse drug events and medication-related harms, including functional impairment, falls and hospitalisation [3–6]. Polypharmacy, commonly defined as the concurrent use of five or more medications [7], multimorbidity and adverse drug reactions are common in the older population, consequently placing them at increased risk of prescribing cascades, and further complicating clinicians’ ability to distinguish between symptoms of a new medical condition and an adverse drug reaction [4, 8].

Transitions of care between care settings [e.g. hospital, community and long-term care facilities (LTCFs)] are associated with an increased risk of medication-related harms including medication errors and adverse events [9]. In 2017, the World Health Organisation launched the Global Patient Safety Challenge on Medication Safety, identifying medication-related harm during transitions of care as one of the three global priority action areas [10]. Transition to LTCFs is associated with an increase in medication use and medication regimen changes [11, 12], heightening risk of medication-related harms and reduced quality of life [1, 13, 14]. As polypharmacy continues to rise internationally [15], together with increasing ageing populations and more individuals entering LTCFs each year [16], judicious prescribing to minimise inappropriate medication use, including prescribing cascades, and the risks of medication-related harms during this critical period is essential [17].

Most research to date has examined prescribing cascades in community-dwelling adults and studies investigating prescribing cascades in the older population, specifically in LTCFs remain sparse [18, 19]. A recent literature review identified 24 common prescribing cascades from 101 studies, but only 15 studies focused on individuals aged ≥65 years and none conducted in people living in LTCFs [18]. This leaves a significant gap in our understanding of prescribing cascades among older individuals residing in LTCFs and during this critical care transition, despite their potential for serious consequences. Additionally, over half of those entering LTCF have dementia [20], adding further complexity to risks of potentially inappropriate prescribing [21]. This study aims to address these gaps by conducting a comprehensive examination of clinically important prescribing cascades before and after entry to LTCFs, and to examine the impact of dementia on these prescribing cascades.

## Methods

### Study design, data source and setting

A population-based retrospective cohort study was conducted using the National Historical Cohort of the Registry of Senior Australians (ROSA) [22]. Briefly, ROSA is a national data platform that has integrated aged care (i.e. assessments and records of nursing homes, home care supports subsidised by the Australian government), health care and social welfare data for ~3.85 million Australians who have accessed aged care services between 2002–2022. The original ROSA datasets used in this study were available from 1 April 2014 to 30 June 2020, due to data availability and included the national aged care eligibility assessments for aged care services (at home or LTCFs), assessments at entry into LTCFs, episodes of care, national pharmaceutical dispensing records (Pharmaceutical Benefits Scheme, includes all medicines subsidised by the Australian Government) and death records (from the National Death Index) [22]. In Australia, universal health care and government subsidies for aged care services exist, with the majority of people accessing services through these programs included in the employed datasets [23, 24]. This study was approved by the University of South Australia, Australian Institute of Health and Welfare, South Australian Department for Health and Wellbeing, New South Wales Population and Health Services, and Department of Defence and Veterans’ Affairs Human Research Ethics committees. A waiver of informed consent was obtained for this study as it only uses deidentified data. This study followed the Strengthening the Reporting of Observational Studies in Epidemiology (STROBE) reporting guideline (Appendix S1) [25].

### Study cohort

The study cohort comprised individuals who entered LTCFs between 1 January 2016 and 31 December 2018, and aged ≥65 years at entry. Aboriginal or Torres Strait Islander people were excluded due to required ethical, governance and leadership by these individuals to undertake analysis of their data in accordance with Indigenous data sovereignty principles [26]. Those identified as palliative according to their care needs assessment at entry to LTCFs (≤90 days after entry) were excluded. Individuals included in the study needed to be dispensed 2 or more medications in the 18 months before and after LTCF entry to enable opportunity for examining prescribing cascades.

### Identification of prescribing cascades

A prescribing cascade involves an initial medication (i.e. index medication) suspected of causing an adverse drug reaction (ADR) and initiation of a medication to treat the ADR (i.e. marker medication) in a patient receiving incident dispensing of the two medications.

A total of 143 unique prescribing cascades were examined in the current study, identified from a 2022 published systematic review [18], 3 scoping reviews [19, 27, 28] and a 2025 international expert consensus lists of clinically important prescribing cascades [29] (Appendix S2).

Prescribing cascades were examined in the 18 months before and after LTCF entry. For each prescribing cascade, the dispensing of the index and marker medication needed to occur within a 12-month period. A 90-day washout period (defined as no prior use of the index or marker medication) was applied to ascertain incident dispensing of either the index or marker medications cascades before and after LTCF entry (Appendix S3).

### Covariates

Characteristics of the study cohort were identified at LTCF entry and included resident characteristics: sex, age, country of birth, preferred language, number of health conditions (identified using Rx-Risk pharmaceutical-based comorbidity index [30] calculated in the 6 months prior to LTCF entry) and presence of dementia (ascertained using recorded diagnosis within aged care assessments datasets and the pharmaceutical-based comorbidity index Rx-Risk) [20]. LTCF characteristics included: location (state, geographical remoteness) [31] and ownership (not for profit, for profit or government).

### Statistical analyses

Descriptive statistics using percentages, and medians and interquartile ranges for continuous variables were employed to summarise the demographic and clinical characteristic of the study cohort. Prescribing cascades were examined using prescription sequence symmetry analyses (PSSA), an established method that assesses the association between two medicines [32]. PSSA is a case-only design that examines the relative timing of an index medication and marker medication. In the absence of a causal relationship, the probability of a patient receiving the marker medication before or after initiating the index medication should be approximately equal, resulting in a symmetrical distribution of prescribing sequences. However, if the index medication potentially leads to an adverse effect that increases the likelihood of subsequent treatment with the marker medication, an asymmetry in prescribing sequences is expected [32]. Using PSSA, the crude sequence ratio (cSR) was calculated for each examined prescribing cascade (within a 12-month exposure window between the index and marker medications). To adjust for underlying prescribing trends, an adjusted sequence ratio (aSR) and 95% confidence interval (95% CI) were calculated. The primary analyses examined prescribing cascades in the overall LTCF population. Secondary analyses were then stratified by dementia status to explore whether the findings differed for people with and without dementia. Sensitivity analyses were performed where the exposure windows were 30-day incremental periods from 30 days to 360 days.

All data preparation and analyses were conducted using SAS 9.4 (SAS Institute, Cary, NC, USA).

## Results

The cohort comprised of 167 850 individuals in 2771 LTCFs nationally (Appendix S4). At LTCF entry, 60.4% (*N* = 101 441) female, with a median age of 86 years [interquartile range (IQR) 80–90], a median of 5 (IQR 4–7) comorbid conditions and 49.4% (*n* = 82 897) were living with dementia after entry to LTCF (Table 1). Most residents were in LTCFs in metropolitan areas (*n* = 115 678, 69.0%) and 53.7% (*n* = 90 071) lived in not-for-profit LTCFs (Table 1). There were 61 unique statistically significant positive prescribing cascades identified (42% of all analysed), of which 28 018 individuals (16.7%) were involved in at least 1 prescribing cascade before LTCF entry and 42 101 (25.1%) after LTCF entry (Table 1).

**Table 1:** Baseline characteristics of study cohort at LTCF entry.

	Study cohort	Before LTCF entry		After LTCF entry	
Characteristics	Total, *n* (%)	At least 1 significant cascade, *n* (%)	No significant cascade, *n* (%)	At least 1 significant cascade, *n* (%)	No significant cascade, *n* (%)
**Total, *n***	167 850	28 018 (16.7)	139 832 (83.3)	42 101 (25.1)	125 749 (74.9)
**Sex**					
Female	101 441 (60.4)	16 714 (59.7)	84 727 (60.6)	25 542 (60.7)	75 899 (60.4)
Male	66 409 (39.6)	11 304 (40.3)	55 105 (39.4)	16 559 (39.3)	49 850 (39.6)
**Age years, median (IQR)**	86 (80–90)	85 (79–89)	86 (80–90)	85 (80–90)	86 (80–90)
65–70 years	8413 (5.0)	1706 (6.1)	6707 (4.8)	2356 (5.6)	6057 (4.8)
71–75	12 928 (7.7)	2493 (8.9)	10 435 (7.5)	3512 (8.3)	9416 (7.5)
76–80	23 384 (13.9)	4493 (16.0)	18 891 (13.5)	6065 (14.4)	17 319 (13.8)
81–85	37 828 (22.5)	6722 (24.0)	31 106 (22.3)	9721 (23.2)	28 107 (22.4)
86–90	48 127 (28.7)	7605 (27.1)	40 522 (29.0)	11 783 (28.0)	36 344 (28.9)
91–95	29 893 (17.8)	4127 (14.7)	25 766 (18.4)	7090 (16.8)	22 803 (18.1)
96+	7277 (4.3)	872 (3.1)	6405 (4.6)	1574 (3.7)	5703 (4.5)
**Country of birth**					
Australia	114 437 (68.2)	18 880 (67.4)	95 557 (68.3)	27 683 (65.8)	86 754 (69.0)
Overseas	52 914 (31.5)	9039 (32.3)	43 875 (31.4)	14 272 (33.9)	38 642 (30.7)
Unknown	499 (0.3)	99 (0.4)	400 (0.3)	146 (0.4)	353 (0.3)
**Preferred language**					
English	151 939 (90.5)	25 256 (90.1)	126 683 (90.6)	37 571 (89.2)	114 368 (91.0)
Other	15 097 (9.0)	2614 (9.3)	12 483 (8.9)	4313 (10.2)	10 784 (8.6)
Unknown	814 (0.5)	148 (0.5)	666 (0.5)	217 (0.5)	597 (0.5)
^a^ **Rx-Risk comorbidity index**					
0–3 (n conditions)	41 436 (24.7)	3088 (11.0)	38 348 (27.4)	10 997 (26.1)	30 439 (24.2)
4–5	47 473 (28.3)	6476 (23.1)	40 997 (29.3)	11 984 (28.5)	35 489 (28.2)
6–10	73 740 (43.9)	16 532 (59.0)	57 208 (40.9)	17 964 (42.7)	55 776 (44.4)
11+	5201 (3.1)	1922 (6.9)	3279 (2.3)	1156 (2.8)	4045 (3.2)
**Dementia before LTCF**	47 880 (28.5)	8073 (28.8)	39 807 (28.5)	–	–
**Dementia after LTCF**	82 897 (49.4)	–	–	21 073 (50.1)	61 824 (49.2)
**Mortality within 12 months of LTCF entry**	48 087 (28.6)	9534 (34.0)	38 553 (27.6)	11 734 (28.0)	36 353 (28.9)
**Number of LTCFs, *n***	2771	2691	NC	2725	NC
**LTCF state**					
Australian Capital Territory	2383 (1.4)	329 (1.2)	2054 (1.5)	552 (1.3)	1831 (1.5)
New South Wales	55 784 (33.2)	8084 (28.9)	47 700 (34.1)	13 608 (32.3)	42 176 (33.5)
Northern Territory	278 (0.2)	34 (0.1)	244 (0.2)	93 (0.2)	185 (0.2)
Queensland	31 487 (18.8)	5685 (20.3)	25 802 (18.5)	7628 (18.1)	23 859 (19.0)
South Australia	14 946 (8.9)	2391 (8.5)	12 555 (9.0)	3814 (9.2)	11 132 (8.9)
Tasmania	4477 (2.7)	678 (2.4)	3799 (2.7)	1033 (2.5)	3444 (2.7)
Victoria	44 575 (26.6)	8233 (29.4)	36 342 (26.0)	12 274 (29.2)	32 301 (25.7)
Western Australia	13 920 (8.3)	2584 (9.2)	11 336 (8.1)	3099 (7.4)	10 821 (8.6)
**Remoteness**					
Major city	115 678 (69.0)	19 600 (70.0)	96 078 (68.7)	29 836 (70.9)	85 842 (68.3)
Inner regional	38 221 (22.8)	6220 (22.2)	32 001 (22.9)	9146 (21.7)	29 075 (23.1)
Outer regional	12 810 (7.6)	2022 (7.2)	10 788 (7.7)	2875 (6.8)	9935 (7.9)
Remote/very remote	688 (0.4)	101 (0.47)	587 (0.4)	144 (0.3)	544 (0.4)
Unknown	453 (0.3)	75 (0.3)	378 (0.3)	100 (0.2)	353 (0.3)
**LTCF provider type**					
Government	6820 (4.1)	1167 (4.2)	5653 (4.0)	1730 (4.11)	5090 (4.1)
Not-for-profit	90 071 (53.7)	14 511 (51.8)	75 560 (54.0)	21 872 (51.95)	68 199 (54.2)
Private	70 959 (42.3)	12 340 (44.0)	58 619 (41.9)	18 499 (43.94)	52 460 (41.7)

Abbreviations: IQR, interquartile range; LTCF, long-term care facility

NC: Not calculated because of double counting. For example, a facility can have people with and without a significant cascade.

a Rx-Risk pharmaceutical-based comorbidity index calculated in the 6 months prior to study entry.

### Prescribing cascades before LTCF entry

**Figure 1:**
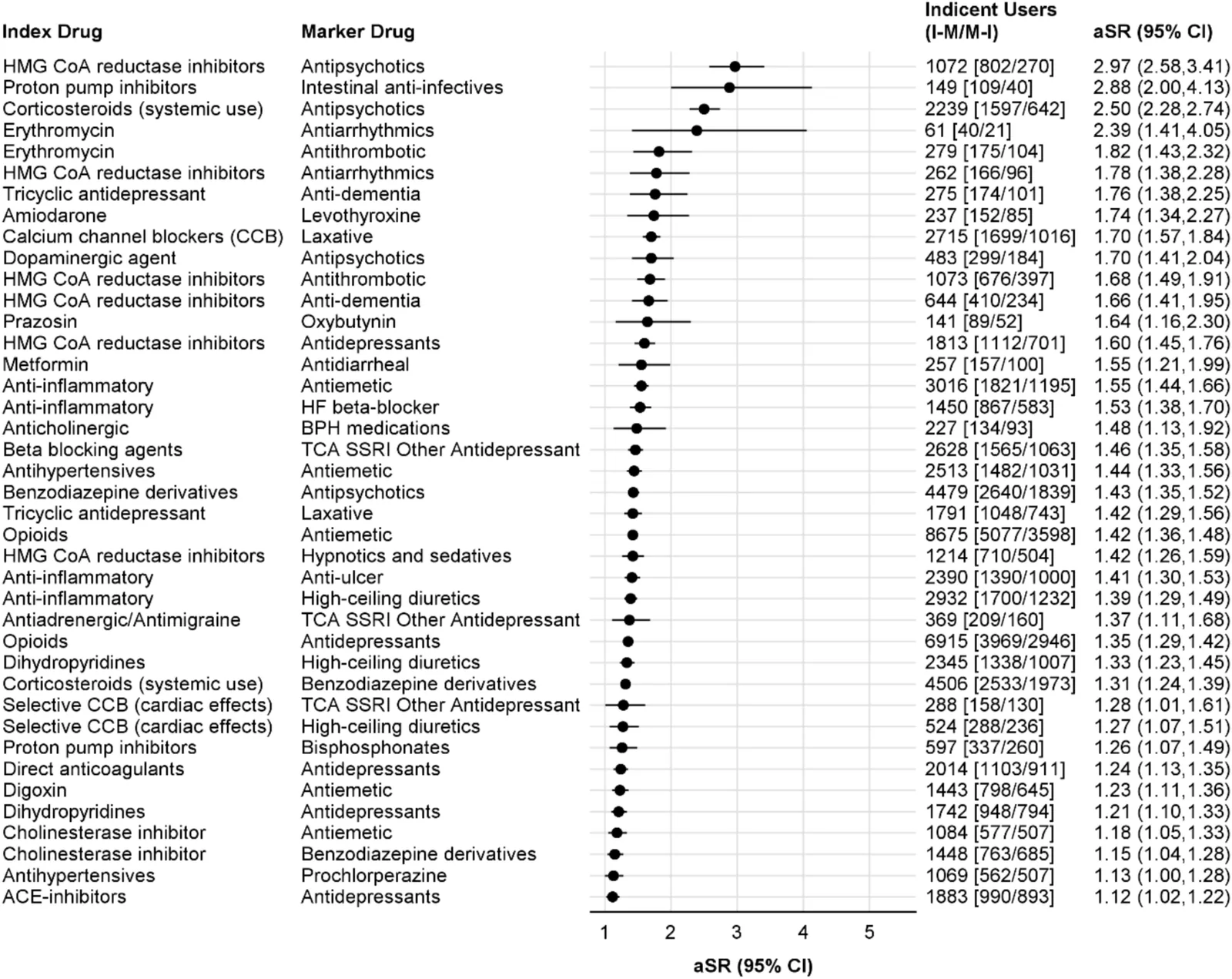
Forest plot of statistically significant prescribing cascades (*n* = 40) during the 18 months before LTCF transition (ordered by adjusted sequence ratio).

In the period before LTCF entry, 40 statistically significant prescribing cascades were identified (Figure 1, see Appendix S5). Initiation of a statin was associated with 6 prescribing cascades, including one third of the top 15 strongest observed associations, ranging from a three-fold increase in prescribing of an antipsychotic for confusion (aSR 2.97, 95% CI 2.58–3.41, *n* = 1072) to a 42% increased likelihood of prescribing of hypnotics and sedatives (aSR 1.42, 95% CI 1.26–1.59, *n* = 1214). Initiation of opioids associated with antiemetic prescribing (for nausea/dizziness) had the highest number of incident users, occurring in 5% of the study cohort (aSR 1.42, 95% CI 1.36–1.48, *n* = 8675), followed by initiation of opioids associated with antidepressants (aSR 1.35, 95% CI 1.29–1.42, *n* = 6915). Among cascades with at least 2000 incident users, initiation of a systemic corticosteroid leading to antipsychotic prescribing was the strongest association (aSR 2.50, 95% CI: 2.28–2.74, *n* = 2239), followed by initiation of calcium channel blockers leading to laxative prescribing (aSR 1.70, 95% CI: 1.57–1.84, *n* = 2715). The most common potential adverse event in the significant prescribing cascades was depression leading to antidepressant prescribing as the marker medication, occurring in 20% (*n* = 8/41) significant prescribing cascades before entry to LTCF, followed by initiation of an antiemetic medication (*n* = 6/41, 15%).

### Prescribing cascades after LTCF entry

**Figure 2:**
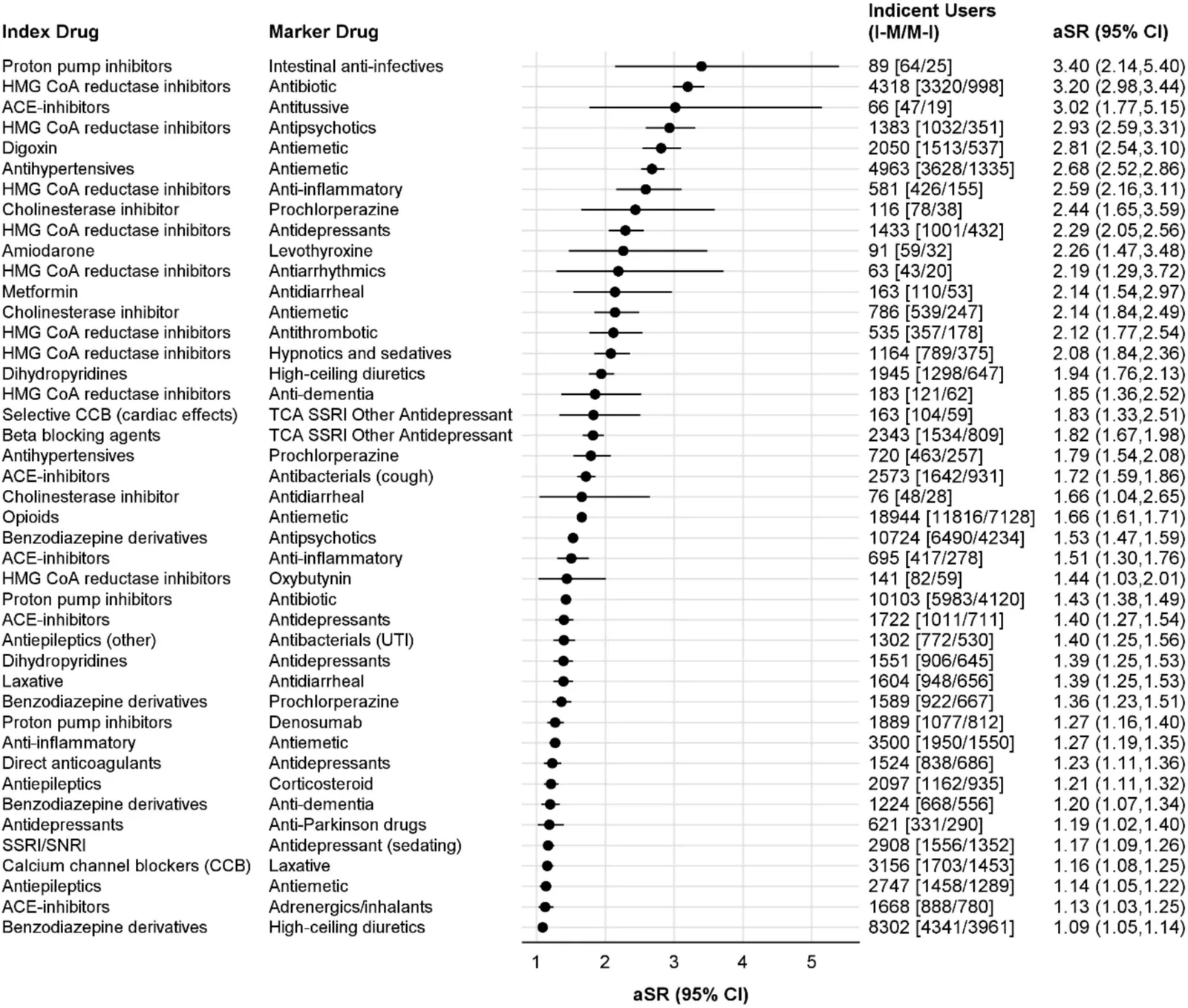
Forest plot of statistically significant prescribing cascades (*n* = 43) during the 18 months after LTCF transition (ordered by adjusted sequence ratio).

Following LTCF entry, 43 statistically significant prescribing cascades were identified (Figure 2, see Appendix S6). Initiation of a statin was associated with nine prescribing cascades (20%) with seven of these having an aSR >2.00, ranging from a three-fold increase in prescribing of an antibiotic for soft-tissue infection (aSR 3.20, 95% CI 2.98–3.44, *n* = 4318) to a two-fold increased likelihood of prescribing of hypnotics and sedatives for sleeplessness (aSR 2.08, 95% CI 1.84–2.36, *n* = 1164). Prescribing cascades with the highest number of incident users included initiation of an opioid associated with prescribing of an antiemetic for nausea/dizziness (aSR 1.66, 95% CI 1.61–1.71, *n* = 18 944), followed by initiation of a benzodiazepine associated with prescribing of an antipsychotic for paradoxical agitation (aSR 1.53, 95% CI 1.47–1.59, *n* = 10 724) and prescribing of an antibiotic for pneumonia following initiation of a proton pump inhibitor (aSR 1.43, 95% CI 1.38–1.49, *n* = 10 103)—for all of these cascades, the number of incident users had at least doubled when compared to before LTCF entry. The most common potential adverse event in the significant prescribing cascades was nausea/dizziness, occurring in 18% (*n* = 8/44) of significant prescribing cascades after entry to LTCF, followed by initiation of an antidepressant medication (*n* = 6/44, 14%).

Appendix S7 shows the prescribing cascades that were not statistically significant for the periods before and after LTCF entry.

### Comparing prescribing cascades before and after LTFC entry

Twenty-three of the statistically significant prescribing cascades occurred both before and after entry to LTCF (Appendix S8). Of these prescribing cascades, 11 associations were significantly higher in individuals after they entered LTCF compared to before LTCF entry, for example, antihypertensives resulting in prescribing of antiemetics (aSR 2.68, 95% CI 2.52–2.86 vs. aSR 1.44, 95% CI 1.33–1.56) and initiation of statins and prescribing of antidepressants (aSR 2.29, 95% CI 2.05–2.56 vs. aSR 1.60, 95% CI 1.45–1.76).

There were 17 prescribing cascades only observed before individuals entered LTCF, with the most common index medications being opioids followed by initiation of antidepressants. There were also 20 prescribing cascades unique to after LTCF entry, with the most common index medications being proton pump inhibitors followed by the initiation of antibacterials and benzodiazepines followed by the initiation of diuretics.

### The impact of dementia on the prescribing cascades

Prior to LTCF entry, 16.9% (*n* = 8073/47 880) of people living with dementia had at least 1 statistically significant prescribing cascade and 16.6% (*n* = 19 945/119 970) among those not living with dementia. Overall, 30 statistically significant prescribing cascades were common to individuals living with dementia and 35 among those not living with dementia (Appendix S9). Six of the prescribing cascades were only identified for those living with dementia and mostly were associated with insomnia (e.g. cholinesterase inhibitors leading to benzodiazepines, aSR 1.16, 95% CI 1.04–1.29, *n* = 1352 and selective serotonin reuptake inhibitors (SSRI)/serotonin-norepinephrine reuptake inhibitors (SNRI) leading to sedating antidepressants, aSR 1.17, 95% CI 1.01–1.36, *n* = 685). See Appendix S10 for a summary comparing cascades among those with and without dementia.

Following LTCF entry, 25.4% (*n* = 21 073/82 897) of people living with dementia had at least 1 statistically significant prescribing cascade and 25.0% (*n* = 21 028/84 953) among those not living with dementia. Overall, 40 statistically significant prescribing cascades occurred in individuals living with dementia and 35 among those not living with dementia (Appendix S11). Thirteen of the cascades were identified only among those living with dementia with the most common being initiation of benzodiazepines leading to high ceiling diuretics (aSR 1.32, 95% CI 1.24–1.40, *n* = 4147) followed by initiation of SSRI/SNRI leading to sedating antidepressants (aSR 1.28, 95% CI 1.16–1.42, *n* = 1437) and antiepileptics leading to antiemetic (aSR 1.38, 95% CI 1.24–1.53, *n* = 1434). Initiation of non-steroidal anti-inflammatory drugs (NSAIDs) leading to antiemetics for nausea was the only cascade significantly higher among those living with dementia (aSR 1.50, 95% CI 1.36–1.67) compared to those without dementia (aSR 1.12, 95% CI 1.03–1.22). Whereas the associations for the initiation of benzodiazepines and statins leading to antipsychotics were significantly higher among those without dementia. See Appendix S12 for a summary comparing cascades among those with and without dementia.

### Sensitivity analyses

**Figure 3:**
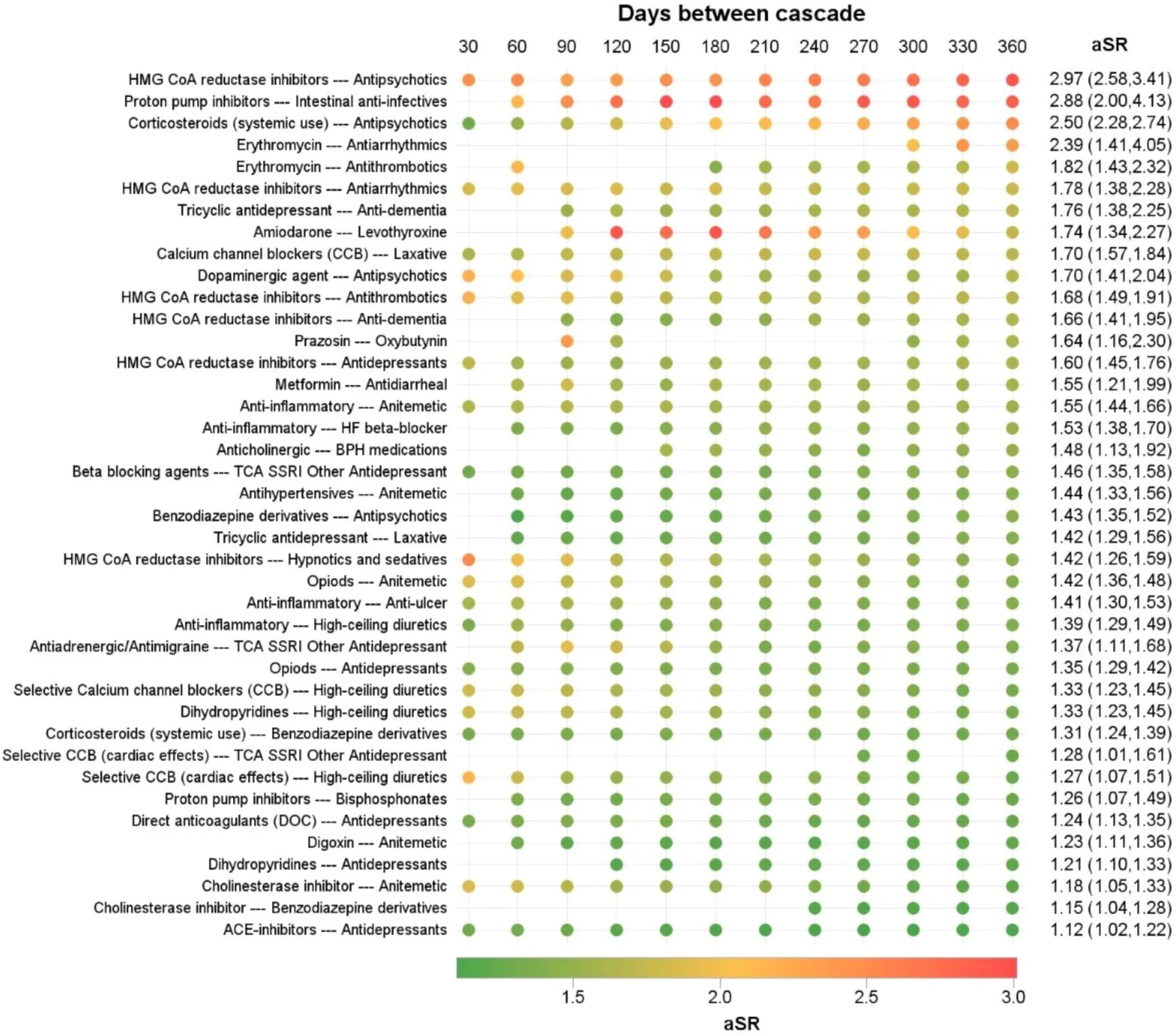
Sensitivity analyses for the period before entry to LTCF. Adjusted sequence ratios using exposure windows (time between medications in the cascade), based on 30-day incremental periods from 30 days to 360 days. Each dot represents the statistically significant aSR based on incremental exposure windows for each cascade where the colours represent the strength of the aSR. Note: The column on the right side of the figure presents the aSR from the main results (e.g., a 365-day window) that are presented in Figure 1.

**Figure 4:**
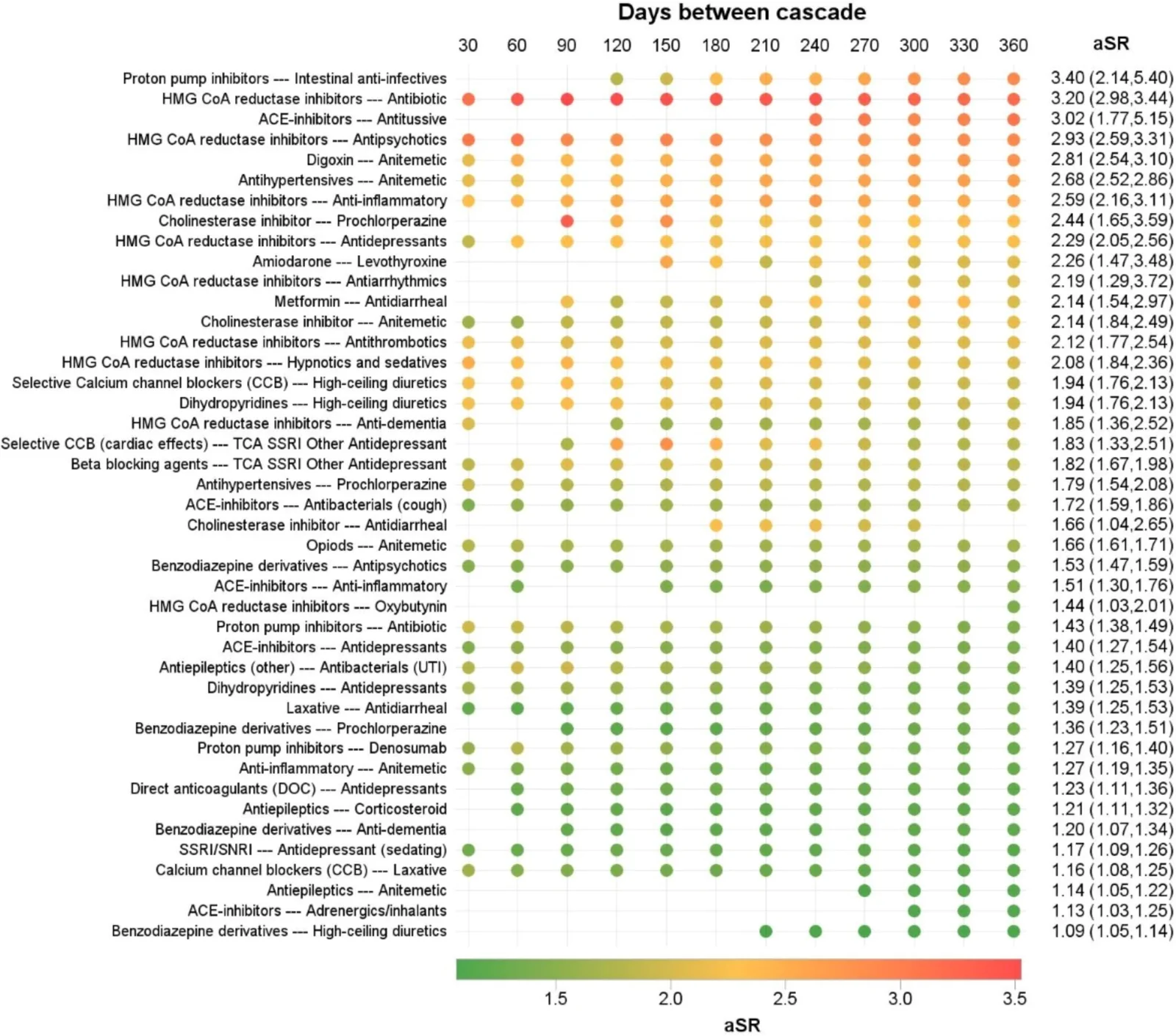
Sensitivity analyses for the period after entry to LTCF. Adjusted sequence ratios using exposure windows (time between medications in the cascade), based on 30-day incremental periods from 30 days to 360 days. Each dot represents the statistically significant aSR based on incremental exposure windows for each cascade where the colours represent the strength of the aSR. Note: The column on the right side of the figure presents the aSR from the main results (e.g., a 365-day window) that are presented in Figure 2.

Of the 40 statistically significant cascades in the period before LTCF entry, 21 cascades were statistically significant using the 30-day exposure window, 31 cascades were significant using the 60-day exposure window and 34 were significant using the 90-day exposure window (Figure 3). For the period after LTCF entry, of the 43 statistically significant cascades, 26 cascades were statistically significant using the 30-day exposure window, 28 cascades were significant using the 60-day exposure window and 32 were significant using the 90-day exposure window (Figure 4).

## Discussion

This large, national cohort study is the first to systematically examine prescribing cascades before and after entry into LTCFs to identify prescribing patterns with potentially harmful consequences for the older population. Of the 61 unique cascades identified, there were 40 positive prescribing cascades prior to LTCF entry (among 16.7% of the study population) and 43 post-entry (among 25.1% of the study population), with 24 occurring across both periods. Our findings highlight the prevalence of prescribing patterns consistent with potential prescribing cascades in this vulnerable population during a critical care transition period. Over half occurred within a 30-day exposure period and three-quarters within 90 days, highlighting the plausibility of prescribing in response to an acute ADR.

Over half of the 61 unique prescribing cascades identified in this study involved medications included in the Beers Criteria as potentially inappropriate medications for older adults [33]. For example, benzodiazepines, antipsychotics and proton pump inhibitors are known to be consistently associated with higher risks of cognitive decline, falls and infections in frail populations [34–36]. Their repeated appearance in cascades both before and after LTCF entry suggests a persistent pattern of potentially harmful prescribing and clear targets for deprescribing. Additionally, cascades involving diuretics following NSAIDs or calcium channel blockers, and sedatives following antidepressants, were also identified and reflect well-recognised pathways by which pharmacological risk can escalate [18, 37].

Many of our findings are consistent with a list of potentially inappropriate prescribing cascades recently developed by an international expert panel [29], including a range of index medications such as ACE-inhibitors, antidepressants, beta-blockers, benzodiazepines, cholinesterase inhibitors and statins. Furthermore, many of our results are consistent with prior literature that examined prescribing cascades [19, 38–40], including amiodarone-induced hypothyroidism, ACE-inhibitor-related cough and multiple statin-associated cascades such as confusion treated with antipsychotics, cognitive impairment treated with anti-dementia medications, and depression treated with antidepressants. Less commonly known side effects associated with statins were also observed including increased infection risk, skin and soft tissue infection (antibiotic prescribing) and depressive symptoms [41, 42]. However, alternative explanations for some observed prescribing associations may include shared underlying pathology, multimorbidity clustering, frailty and increased infection risk after LTCF entry.

Our study adds new evidence by evaluating the temporal relationship of prescribing cascades surrounding LTCF entry, a transition period often associated with medication regimen changes. Of the prescribing cascades persistent across both periods, the strength of the association was significantly stronger after LTCF entry for the cascades of beta blocking agents and statins leading to the subsequent initiation of antidepressants, and calcium channel blockers leading to subsequent initiation of diuretics. Additionally, there were 20 prescribing cascades appearing only after LTCF entry, with the most common index medications being ACE-inhibitors, statins and proton pump inhibitors followed by the initiation of antibacterials. Also highlighting the changes in prescribing patterns after entry to LTCF was the two-fold increase in the number of incident users for the cascade of benzodiazepines leading to the subsequent initiation of antipsychotics. This increase across the transition period for these medications have been reported previously [11]. These findings may also reflect changes in care setting, resulting in increased clinical monitoring, changes in residents health status, increased frailty or differences in symptom detection and prescribing.

We identified 13 prescribing cascades among those with dementia that occurred after LTCF entry. For example, the initiation of SSRI/SNRIs was associated with subsequent initiation of sedating antidepressants, which may reflect the emergence or worsening of behavioural and psychological symptoms of dementia, including agitation, insomnia or restlessness, which are often managed with sedating agents [43]. In support of this finding, individuals with dementia are more likely to experience adverse neuropsychiatric effects from SSRIs, including sleep disturbances, which may lead to additional psychotropic prescribing in residential settings [43, 44]. Also, among those with dementia, the initiation of antipsychotics and antidepressants leading to subsequent initiation of tertiary amines/dopaminergics only occurred after LTCF entry. The association between the use of antipsychotics and drug-induced parkinsonism is well known [43, 45] and usually reversible upon cessation of the antipsychotic. It is therefore concerning that 408 people were exposed to this prescribing cascade, which is avoidable in most cases. The association between use of antidepressants and movement disorders is less well established; however, studies have identified this potential association, supporting our findings [46, 47]. These dementia-related cascades are important in LTCFs because they involve medications commonly used in dementia care and may provide opportunities for earlier recognition of adverse effects, medication review, deprescribing and the use of non-drug strategies where appropriate.

Our findings highlight the importance of recognising prescribing cascades as a critical contributor to potentially inappropriate medication use in long-term care. Several of the cascades identified in this study involved medication classes already known to be high-risk in older populations, such as antipsychotics, diuretics, opioids and sedatives. The frequent involvement of these medications in cascades suggests that their use should be carefully reviewed, particularly when prescribed shortly after another new medication. Furthermore, some of the observed sequences may represent avoidable prescribing cascades, particularly when a known adverse effect appears to be treated with another high-risk medication.

No studies to date have comprehensively assessed cascade patterns both before and after LTCF entry. This gap is particularly significant given the elevated risk of ADRs during care transitions and the high prevalence of polypharmacy in aged care [48]. Our study addresses this critical gap by systematically evaluating 143 potential prescribing cascades in a large, national cohort of Australian LTCF residents, using robust administrative data and stratifying results by dementia status. By examining cascade patterns across the LTCF transition, our findings provide insights into high-risk prescribing pathways and offer new opportunities for targeted medication review and deprescribing initiatives in this vulnerable population.

Several limitations should be considered. First, PSSA identifies temporal asymmetry and cannot confirm that an ADR occurred, or that the marker medication was prescribed in response to such an event. However, our analyses focused on prescribing cascades previously reported in the literature, many of which involve marker medications with well-established roles in managing ADRs associated with the index medications. In addition, our sensitivity analyses highlighted the temporality of the prescribing cascades, reducing the potential for temporal misclassification, especially for chronic or slowly evolving ADRs by comparison to acute or subacute ADRs that are likely to occur within months. Second, administrative dispensing data lack information about symptom severity and prescribing intent and therefore limiting interpretation of inappropriate care. Third, mortality within 12 months of LTFC entry was substantial, which may introduce survivor bias. Finally, our findings are based on Australian administrative data and may not be directly generalisable to all aged care systems.

In this population-based study of LTCF residents, we identified prescribing cascades involving high-risk medications, both before and after entry into care. These findings highlight the need for proactive medication reviews and deprescribing strategies to mitigate potential medication-related harm. Addressing prescribing cascades at key transition points may improve the safety and quality of medication-related care for the older population worldwide.

## Supplementary Material

Supplementary_materials_afag166

## Data Availability

The data from this study is not available for sharing by the researchers due to ethical restrictions and approvals required to access and link the study data. The datasets can be recreated by seeking approval for access from original data custodians for all datasets included in the existing data source and data integrating authorities to link it.
