# Correction to: Prescribing cascades potentially associated with harms before and after transition to long-term care facilities

**DOI:** 10.1093/ageing/afag308

**Published:** 2026-10-15

This is a correction to: Craig Hansen, Lisa Kalisch Ellett, Tracy Air, Maria C Inacio, Gillian Elizabeth Caughey, Prescribing cascades potentially associated with harms before and after transition to long-term care facilities, *Age and Ageing*, Volume 55, Issue 6, June 2026, afag166, https://doi.org/10.1093/ageing/afag166

In the originally published version of this manuscript, there was an error in the second co-author’s name. This has been corrected to read “Lisa Kalisch Ellett”.

