# Supplementary material for: Prescribing cascades potentially associated with harms before and after transition to long-term care facilities
Source: Age Ageing. 2026 Jun 18;55(6):afag166. doi: 10.1093/ageing/afag166 (PMC13278772; doi:10.1093/ageing/afag166)
Supplement: Supplementary_materials_afag166 [file supplementary_materials_afag166.docx]

**ONLINE SUPPLEMENTAL MATERIALS**

Prescribing Cascades Potentially Associated with Harms Before and After Transition to Long-Term Care Facilities.

**Appendix 1**: STROBE Statement—Checklist of items that should be included in reports of cohort studies.

**Appendix 2:** Prescribing cascades analysed in the study (ordered by Index ATC).

**Appendix 3:** PSSA study design and time periods for incident medication dispensing for before and after LTCF entry (Figure).

**Appendix 4:** Flow diagram for participant inclusion. LTCF: Long-term care facility. ATSI: Aboriginal and Torres Strait Island. ATC: anatomical therapeutic chemical**.**

**Appendix 5:** Statistically significant prescribing cascades (N = 40) during the 18 months before LTCF transition (ordered by adjusted sequence ratio).

**Appendix 6:** Statistically significant prescribing cascades (n = 43) during the 18 months after LTCF transition (ordered by adjusted sequence ratio).

**Appendix 7:** Non-statistically significant prescribing cascades during the 18 months before and after LTCF transition (ordered by adjusted sequence ratio).

**Appendix 8:** Summary of statistically significant prescribing cascades occurring before and after entry to LTCF.

**Appendix 9:** Statistically significant prescribing cascades during the 18 months before entering LTCF, stratified by dementia status (ordered by adjusted sequence ratio).

**Appendix 10:** Summary of statistically significant prescribing cascades occurring before entry to LTCF stratified by those with and without dementia.

**Appendix 11:** Statistically significant prescribing cascades during the 18 months after entering LTCF, stratified by dementia status (ordered by adjusted sequence ratio).

**Appendix 12:** Summary of statistically significant prescribing cascades occurring after entry to LTCF stratified by those with and without dementia.

**Appendix 1**: STROBE Statement—Checklist of items that should be included in reports of cohort studies.

|  | Item No | Recommendation | Page No |
| --- | --- | --- | --- |
| **Title and abstract** | 1 | (*a*) Indicate the study’s design with a commonly used term in the title or the abstract | 1 |
|  |  | (*b*) Provide in the abstract an informative and balanced summary of what was done and what was found |  |
| Introduction | | | |
| Background/rationale | 2 | Explain the scientific background and rationale for the investigation being reported | 2 |
| Objectives | 3 | State specific objectives, including any prespecified hypotheses | 3 |
| Methods | | | |
| Study design | 4 | Present key elements of study design early in the paper | 3 |
| Setting | 5 | Describe the setting, locations, and relevant dates, including periods of recruitment, exposure, follow-up, and data collection | 3,4 |
| Participants | 6 | (*a*) Give the eligibility criteria, and the sources and methods of selection of participants. Describe methods of follow-up | 3,4 |
|  |  | (*b*) For matched studies, give matching criteria and number of exposed and unexposed |  |
| Variables | 7 | Clearly define all outcomes, exposures, predictors, potential confounders, and effect modifiers. Give diagnostic criteria, if applicable | 5 |
| Data sources/ measurement | 8* | For each variable of interest, give sources of data and details of methods of assessment (measurement). Describe comparability of assessment methods if there is more than one group | 5 |
| Bias | 9 | Describe any efforts to address potential sources of bias | 6 |
| Study size | 10 | Explain how the study size was arrived at | 4,5 |
| Quantitative variables | 11 | Explain how quantitative variables were handled in the analyses. If applicable, describe which groupings were chosen and why | 5-6 |
| Statistical methods | 12 | (*a*) Describe all statistical methods, including those used to control for confounding | 5-6 |
|  |  | (*b*) Describe any methods used to examine subgroups and interactions |  |
|  |  | (*c*) Explain how missing data were addressed |  |
|  |  | (*d*) If applicable, explain how loss to follow-up was addressed |  |
|  |  | (*e*) Describe any sensitivity analyses |  |
| Results | | |  |
| Participants | 13* | (a) Report numbers of individuals at each stage of study—eg numbers potentially eligible, examined for eligibility, confirmed eligible, included in the study, completing follow-up, and analysed | 6 |
|  |  | (b) Give reasons for non-participation at each stage |  |
|  |  | (c) Consider use of a flow diagram |  |
| Descriptive data | 14* | (a) Give characteristics of study participants (eg demographic, clinical, social) and information on exposures and potential confounders | 6 + Table 1 |
|  |  | (b) Indicate number of participants with missing data for each variable of interest |  |
|  |  | (c) Summarise follow-up time (eg, average and total amount) |  |
| Outcome data | 15* | Report numbers of outcome events or summary measures over time | 7-8 |
| Main results | 16 | (*a*) Give unadjusted estimates and, if applicable, confounder-adjusted estimates and their precision (eg, 95% confidence interval). Make clear which confounders were adjusted for and why they were included | 6-10 |
|  |  | (*b*) Report category boundaries when continuous variables were categorized |  |
|  |  | (*c*) If relevant, consider translating estimates of relative risk into absolute risk for a meaningful time period |  |
| Other analyses | 17 | Report other analyses done—eg analyses of subgroups and interactions, and sensitivity analyses | Supplementary materials |
| **Discussion** |  |  |  |
| Key results | 18 | Summarise key results with reference to study objectives | 11-14 |
| Limitations | 19 | Discuss limitations of the study, taking into account sources of potential bias or imprecision. Discuss both direction and magnitude of any potential bias | 14 |
| Interpretation | 20 | Give a cautious overall interpretation of results considering objectives, limitations, multiplicity of analyses, results from similar studies, and other relevant evidence | 11-14 |
| Generalisability | 21 | Discuss the generalisability (external validity) of the study results |  |
| **Other information** | | | |
| Funding | 22 | Give the source of funding and the role of the funders for the present study and, if applicable, for the original study on which the present article is based |  |

*Give information separately for exposed and unexposed groups.

**Note:** An Explanation and Elaboration article discusses each checklist item and gives methodological background and published examples of transparent reporting. The STROBE checklist is best used in conjunction with this article (freely available on the Web sites of PLoS Medicine at http://www.plosmedicine.org/, Annals of Internal Medicine at http://www.annals.org/, and Epidemiology at http://www.epidem.com/). Information on the STROBE Initiative is available at http://www.strobe-statement.org.

| **Appendix 2:** Prescribing cascades analysed in the study (ordered by Index ATC). | | | | |
| --- | --- | --- | --- | --- |
| **Index ATC** | **Index Name** | **Marker ATC** | **Marker Name** | **Averse Drug Reaction (ADR)** |
| A02BA02 | Ranitidine | C03C | High-ceiling diuretics | Edema peripheral |
| A02BA02 | Ranitidine | C07AB07 C07AG02 C07AB02 C07AB12 | HF beta-blocker | Heart failure |
| A02BC | Proton pump inhibitors | J01 | Antibiotic | Pneumonia |
| A02BC | Proton pump inhibitors | M05BA | Bisphosphonates | Bone loss |
| A02BC | Proton pump inhibitors | M05BX04 | Denosumab | Bone loss |
| A02BC | Proton pump inhibitors | A07A | Intestinal anti-infectives | Clostridium difficile infection |
| A03FA01 | Metoclopramide | N04 | Anti-Parkinson drugs | Parkinsonism |
| A06A | Laxative | A07 | Antidiarrheal | Diarrhea |
| A10BA02 | Metformin | A07 | Antidiarrheal | Diarrhea |
| A10BG | Pioglitazone | C03C | High-ceiling diuretics | Edema peripheral |
| A10BH | Dipeptidyl peptidase 4 (DPP-4) inhibitors | M01A | NSAIDs | Arthritis |
| A10BK | SGLT2-I | D01 | Antifungal | Genital infections |
| A10BK | SGLT2-I | J01C J01D J01E J01M J01X | Antibacterials (UTI) | Urinary tract infection (UTI) |
| A10BK | SGLT2-I | J02AC01 | Antifungal | Genital infections |
| B01AC07 | Dipyridamole | A04AD | Prochlorperazine | Orthostatic hypotension |
| B01AE07 B01AF01 B01AF02 | Direct anticoagulants | N06A | Antidepressants | Depression |
| C01AA05 | Digoxin | A03FA03 N05AD08 A03FA01 A04AD A04AA | Antiemetic | Nausea/dizziness |
| C01BD01 | Amiodarone | H03AA01 | Levothyroxine | Hypothyroidism |
| C01BD01 | Amiodarone | H03BB01 | Carbimazole | Hyperthyroidism |
| C02 C03 C07 C08 C09 | Antihypertensives | A03FA03 N05AD08 A03FA01 A04AD A04AA | Antiemetic | Nausea/dizziness |
| C02 C03 C07 C08 C09 | Antihypertensives | A04AD | Prochlorperazine | Nausea/dizziness |
| C02A N02CX | Antiadrenergic/Antimigraine | N06AA N06AB N06AX | TCA SSRI Other Antidepressant | Depression |
| C02CA01 | Prazosin | A04AD | Prochlorperazine | Orthostatic hypotension |
| C02CA01 | Prazosin | G04BD04 | Oxybutynin | Urinary incontinence |
| C03B C03A | Low-ceiling diuretics | G04BE | Medications for ED | Erectile dysfunction (ED) |
| C03B C03A | Low-ceiling diuretics | M04A | Anti-gout medication | Gout |
| C03C | High-ceiling diuretics | G04BD04 | Oxybutynin | Urinary incontinence |
| C03C | High-ceiling diuretics | G04BE | Medications for ED | Erectile dysfunction (ED) |
| C03C | High-ceiling diuretics | G04C | Benign prostatic hypertrophy | Diuresis excessive |
| C03D | Aldosterone antagonists | G04BE | Medications for ED | Erectile dysfunction (ED) |
| C07 | Beta blocking agents | G04BE | Medications for ED | Erectile dysfunction (ED) |
| C07 | Beta blocking agents | N06AA N06AB N06AX | TCA SSRI Other Antidepressant | Depression |
| C08 | Calcium channel blockers (CCB) | A06A | Laxative | Constipation |
| C08CA | Dihydropyridines | C03C | High-ceiling diuretics | Edema peripheral |
| C08CA | Dihydropyridines | G04BE | Medications for ED | Erectile dysfunction (ED) |
| C08CA | Dihydropyridines | N06A | Antidepressants | Depression |
| C08D | Selective CCB (cardiac effects) | G04BE | Medications for ED | Erectile dysfunction (ED) |
| C08D | Selective CCB (cardiac effects) | C03C | High-ceiling diuretics | Edema peripheral |
| C08D | Selective CCB (cardiac effects) | N06AA N06AB N06AX | TCA SSRI Other Antidepressant | Depression |
| C09A | ACE-inhibitors | R05F R05D | Antitussive | Cough |
| C09A | ACE-inhibitors | J01A J01B J01F J01G J01R | Antibacterials (cough) | Cough |
| C09A | ACE-inhibitors | G04BE | Medications for ED | Erectile dysfunction (ED) |
| C09A | ACE-inhibitors | R06A | Antihistamines (systemic use) | Cough |
| C09A | ACE-inhibitors | M01A | NSAIDs | Arthritis |
| C09A | ACE-inhibitors | R03A | Adrenergics/inhalants | Cough |
| C09A | ACE-inhibitors | N06A | Antidepressants | Depression |
| C09C C09D | Angiotensin II receptor blocker | G04BE | Medications for ED | Erectile dysfunction (ED) |
| C10AA | HMG CoA reductase inhibitors (statins) (statins) | J01 | Antibiotic | Skin soft tissue infection |
| C10AA | HMG CoA reductase inhibitors (statins) | N06A | Antidepressants | Depression |
| C10AA | HMG CoA reductase inhibitors (statins) | P01BC01 | Quinine | Muscle cramps |
| C10AA | HMG CoA reductase inhibitors (statins) | C01B | Antiarrhythmics | Arrhythmia |
| C10AA | HMG CoA reductase inhibitors (statins) | M01A | NSAIDs | Myalgia/myositis |
| C10AA | HMG CoA reductase inhibitors (statins) | N06D | Anti-dementia | Cognitive impairment |
| C10AA | HMG CoA reductase inhibitors (statins) | G04BE | Medications for ED | Erectile dysfunction (ED) |
| C10AA | HMG CoA reductase inhibitors (statins) | B01AA B01AE B01AF | Antithrombotic | Arrhythmia |
| C10AA | HMG CoA reductase inhibitors (statins) | N05C | Hypnotics and sedatives | Insomnia |
| C10AA | HMG CoA reductase inhibitors (statins) | G04BD04 | Oxybutynin | Urinary incontinence |
| C10AA | HMG CoA reductase inhibitors (statins) | N05A | Antipsychotics | Confusion state |
| C10AA | HMG CoA reductase inhibitors (statins) | N06B | Psychostimulants (ADHD) | Somnolence |
| C10BA | Lipid modifying medications | N06B | Psychostimulants (ADHD) | Somnolence |
| G04BD | For urinary frequency and incontinence | C02 C03 C07 C08 C09 | Antihypertensives | Hypertension |
| G04BD10 G04BD04 G04BD08 G04BD07 | Urinary frequency incontinence | N06D | Anti-dementia | Cognitive impairment |
| G04CB02 G04CB01 G04CA02 G04CA52 G04CA01 G04CA04 | Benign Prostatic Hyperplasia | A04AD | Prochlorperazine | Dizziness |
| H02A | Corticosteroids (systemic use) | N05BA N05CD | Benzodiazepine derivatives | Insomnia |
| H02A | Corticosteroids (systemic use) | C02 C03 C07 C08 C09 | Antihypertensives | Hypertension |
| H02A | Corticosteroids (systemic use) | N05A | Antipsychotics | Psychiatric disorders |
| H02A | Corticosteroids (systemic use) | A10B | Blood lowering medications | Diabetes |
| J01A | Tetracyclines | M01A | NSAIDs | Arthritis |
| J01FA01 | Erythromycin | C01B | Antiarrhythmics | Arrhythmia |
| J01FA01 | Erythromycin | B01AA B01AE B01AF | Antithrombotic | Arrhythmia |
| M01A | NSAIDs | A02B | Anti-ulcer | GI symptoms |
| M01A | NSAIDs | C07AB07 C07AG02 C07AB02 C07AB12 | HF beta-blocker | Heart failure |
| M01A | NSAIDs | A03FA03 N05AD08 A03FA01 A04AD A04AA | Antiemetic | Nausea/dizziness |
| M01A | NSAIDs | A04AD | Prochlorperazine | Nausea/dizziness |
| M01A | NSAIDs | C03C | High-ceiling diuretics | Edema peripheral |
| M01A | NSAIDs | C02 C03 C07 C08 C09 | Antihypertensives | Hypertension |
| M01AH | Coxibs | C02 C03 C07 C08 C09 | Antihypertensives | Hypertension |
| M05BA | Bisphosphonates | A02BC | Proton pump inhibitors | Peptic ulcer |
| M05BA | Bisphosphonates | A02A A02BA A02BC A02BX | Medications for acid disorders | Gastrointestinal disorders |
| N02A | Opiods | A03FA03 N05AD08 A03FA01 A04AD A04AA | Antiemetic | Nausea/dizziness |
| N02A | Opioids | A04AD | Prochlorperazine | Nausea/dizziness |
| N02A | Opioids | N06A | Antidepressants | Depression |
| N02BF | Gabapentinoid | C03C | High-ceiling diuretics | Edema peripheral |
| N02BF | Gabapentinoid | A04AD | Prochlorperazine | Nausea/dizziness |
| N03A | Antiepileptics | D07 | Corticosteroid | Rash topical |
| N03A | Antiepileptics | A03FA03 N05AD08 A03FA01 A04AD A04AA | Antiemetic | Nausea/dizziness |
| N03A | Antiepileptics | C03C | High-ceiling diuretics | Edema peripheral |
| N03A | Antiepileptics | H03AA01 | Levothyroxine | Hypothyroidism |
| N03AF01 | Carbamazepine | H03AA01 | Levothyroxine | Hypothyroidism |
| N03AG01 | Valproate | H03AA01 | Levothyroxine | Hypothyroidism |
| N03AX | Antiepileptics (other) | J01C J01D J01E J01M J01X | Antibacterials (UTI) | Urinary tract infection (UTI) |
| N04B | Dopaminergic agent | N05A | Antipsychotics | Paradoxical agitation |
| N05A | Antipsychotics | C02CA01 | Prazosin | Urinary retention |
| N05A | Antipsychotics | G04CB02 G04CB01 G04CA02, G04CA52 G04CA01 G04CA04 | Dutasteride Finasteride Tamsulosin Dutasteride/Tamsulosin Alfuzosin Silodosin | Urinary retention in BPH |
| N05A | Antipsychotics | C07 | Beta blocking agents | Extrapyramidal symptoms |
| N05A | Antipsychotics | N05BA N05CD | Benzodiazepine derivatives | Akathisia or tardive movements |
| N05A | Antipsychotics | C01B | Antiarrhythmics | Arrhythmia |
| N05A | Antipsychotics | B01AA B01AE B01AF | Antithrombotic | Arrhythmia |
| N05A | Antipsychotics | G02CB | Prolactin inhibitors | Hyperprolactinemia |
| N05A | Antipsychotics | N04 | Anti-Parkinson drugs | Parkinsonism/Extrapyramidal symptoms |
| N05A | Antipsychotics | A10 | Blood lowering medications | Diabetes |
| N05AA01 N05AH03 N05AC01 N05AD01 N05AH04 N05AX08 N05AE04 | Antipsychotic (w/Anticohol.) | C02CA01 | Prazosin | Urinary retention |
| N05AA01 N05AH03 N05AC01 N05AD01 N05AH04 N05AX08 N05AE04 | Antipsychotic (w/Anticohol.) | G04CB02 G04CB01 G04CA02, G04CA52 G04CA01 G04CA04 | Dutasteride Finasteride Tamsulosin Dutasteride/Tamsulosin Alfuzosin Silodosin | Urinary retention in BPH |
| N05BA N05CD | Benzodiazepine derivatives | N05A | Antipsychotics | Paradoxical agitation |
| N05BA N05CD | Benzodiazepine derivatives | C03C | High-ceiling diuretics | Edema peripheral |
| N05BA N05CD | Benzodiazepine derivatives | A04AD | Prochlorperazine | Nausea/dizziness |
| N05BA N05CD | Benzodiazepine derivatives | N06D | Anti-dementia | Cognitive impairment |
| N06A | Antidepressants | N02B | Other analgesics/antipyretics | Migraine |
| N06A | Antidepressants | N04 | Anti-Parkinson drugs | Parkinsonism |
| N06A | Antidepressants | G04BD04 | Oxybutynin | Urinary incontinence |
| N06A | Antidepressants | N05C | Hypnotics and sedatives | Insomnia |
| N06A | Antidepressants | N05BA N05CD | Benzodiazepine derivatives | Tremor |
| N06AA | Tricyclic antidepressant | A04AD | Prochlorperazine | Orthostatic hypotension |
| N06AA | Tricyclic antidepressant | N06D | Anti-dementia | Cognitive impairment |
| N06AA | Tricyclic antidepressant | A06A | Laxative | Constipation |
| N06AA | Tricyclic antidepressant | R03A | Adrenergics/inhalants | Shortness of beath |
| N06AA N06AB N06AX | TCA SSRI Other Antidepressant | A10 | Blood lowering medications | Diabetes |
| N06AA N06AB N06AX | TCA SSRI Other Antidepressant | C02 C03 C07 C08 C09 | Antihypertensives | Hypertension |
| N06AB | SSRI | G04BD04 | Oxybutynin | Urinary incontinence |
| N06AX | Lithium | H03AA01 | Levothyroxine | Hypothyroidism |
| N06AX | Lithium | N04 | Anti-Parkinson drugs | Extrapyramidal symptoms |
| N06AX | Lithium | C07AA05 | Propranolol | Tremor |
| N06AX | Lithium | N04 | Anti-Parkinson drugs | Parkinsonism |
| N06AX | Lithium | N05BA N05CD | Benzodiazepine derivatives | Tremor |
| N06AX23 N06AX16 N06AX21 N06AB | SSRI/SNRI | N05BA N05CD | Benzodiazepine derivatives | Insomnia |
| N06AX23 N06AX16 N06AX21 N06AB | SSRI/SNRI | N05CH01 | Melatonin | Insomnia |
| N06AX23 N06AX16 N06AX21 N06AB | SSRI/SNRI | N06AX11 N06AX05 N06AA09 N06AA12 N06AA10 N06AX22 | Antidepressant (sedating) | Insomnia |
| N06D | Anti-dementia | G04BD04 | Oxybutynin | Urinary incontinence |
| N06DA | Cholinesterase inhibitor | G04BD04 | Oxybutynin | Urinary incontinence |
| N06DA | Cholinesterase inhibitor | A04AD | Prochlorperazine | Orthostatic hypotension |
| N06DA | Cholinesterase inhibitor | N05BA N05CD | Benzodiazepine derivatives | Insomnia |
| N06DA | Cholinesterase inhibitor | A03FA03 N05AD08 A03FA01 A04AD A04AA | Antiemetic | Nausea/dizziness |
| N06DA | Cholinesterase inhibitor | A02BC | Proton pump inhibitors | Peptic ulcer |
| N06DA | Cholinesterase inhibitor | A07 | Antidiarrheal | Diarrhea |
| N06DX01 | Memantine | N03 | Antiepileptics | Seizures |
| R03BA | Inhaled corticosteroids | D01A | Topical antifungals | Oral candidiasis |
| R03BA | Inhaled corticosteroids | C03C | High-ceiling diuretics | Edema peripheral |
| R03BA | Inhaled corticosteroids | A02BC | Proton pump inhibitors | Peptic ulcer |
| R03BB | Anticholinergic | C02CA01 | Prazosin | Urinary retention |
| R03BB | Anticholinergic | G04CB02 G04CB01 G04CA02, G04CA52 G04CA01 G04CA04 | Dutasteride Finasteride Tamsulosin Dutasteride/Tamsulosin Alfuzosin Silodosin | Urinary retention in BPH |
| R03BB | Anticholinergic | S01ED02, S01ED01, S01EE | Betaxolol, Timolol, Prostaglandin analogue | Narrow angle glaucoma |
| S01EC04 | Brinzolamide | C03C | High-ceiling diuretics | Edema peripheral |
| S01EE01 | Latanoprost | C03C | High-ceiling diuretics | Edema peripheral |


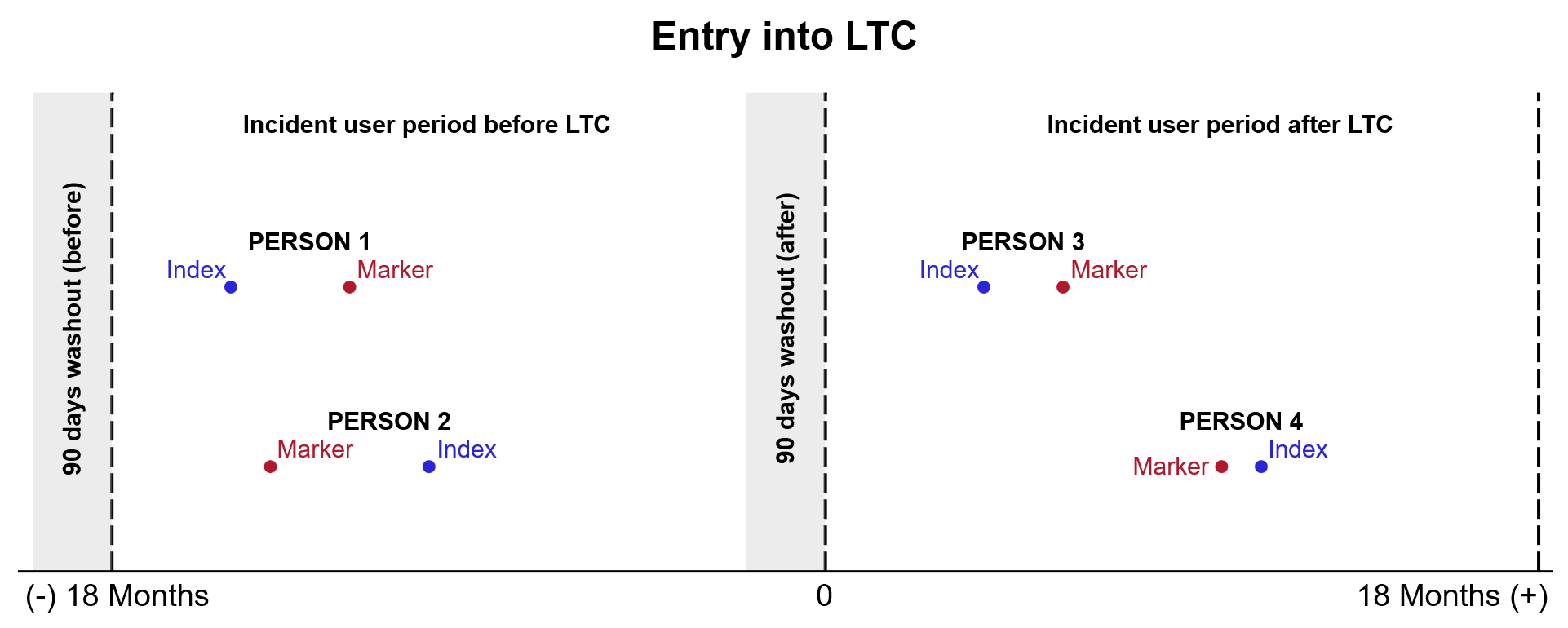


**Appendix 3:** PSSA study design and time periods for incident medication dispensing for before and after LTCF entry.


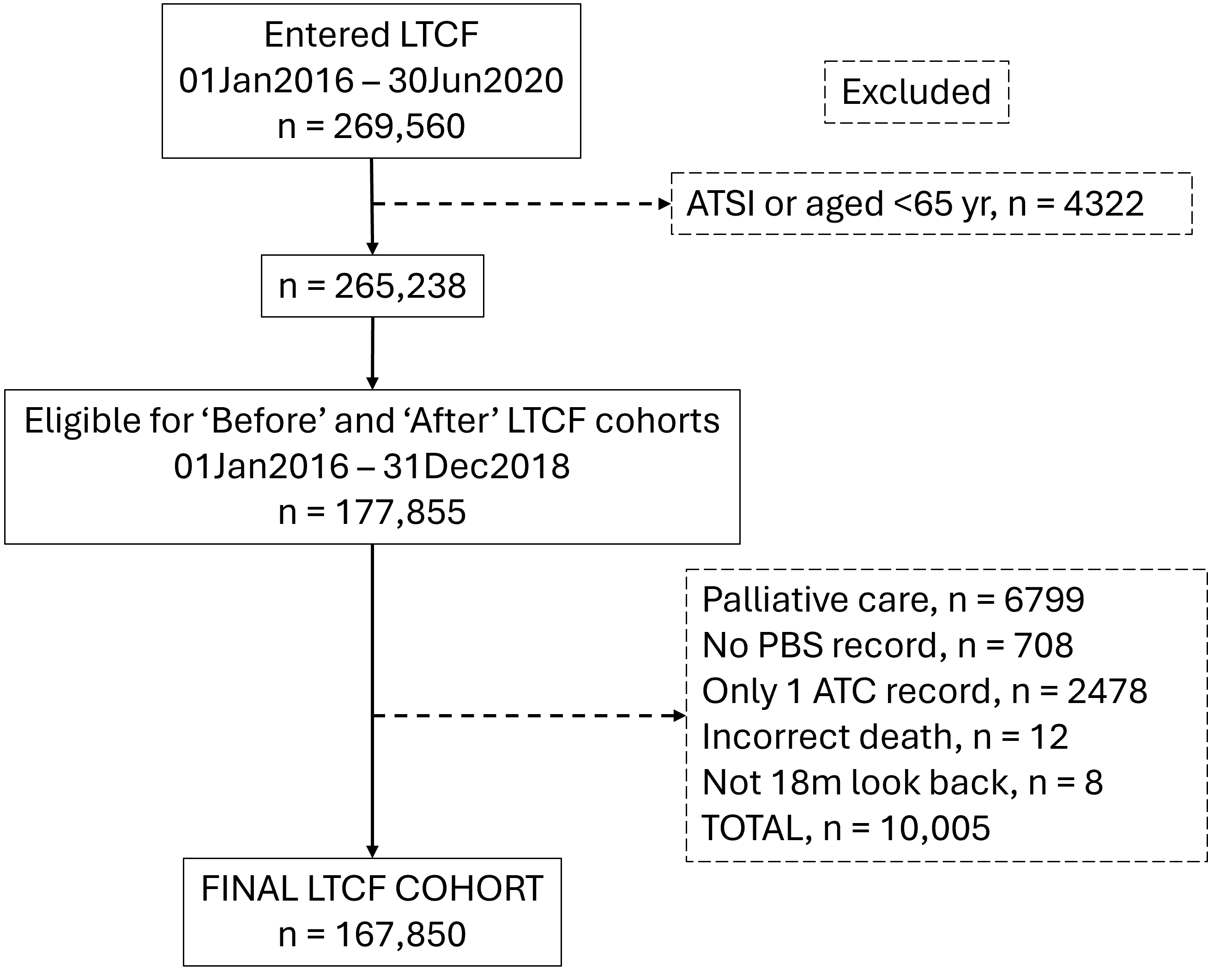


**Appendix 4:** Flow diagram for participant inclusion. LTCF: Long-term care facility. ATSI: Aboriginal and Torres Strait Island. ATC: anatomical therapeutic chemical**.**

| **Appendix 5:** Statistically significant prescribing cascades (N = 40) during the 18 months before LTCF transition (ordered by adjusted sequence ratio). Presented in Figure 1 of the main manuscript. | | | | | |
| --- | --- | --- | --- | --- | --- |
| **Index Medication** | **Potential ADR** | **Marker Medication** | **Incident users**  **[I-M/M-I]** | **Crude SR**  **(95%CI)** | **Adjusted SR (95%CI)** |
| HMG CoA reductase inhibitors (C10AA) (statins) | Confusion state | Antipsychotics (N05A) | 1072 [802/270] | 2.97 (2.59,3.41) | 2.97 (2.58,3.41) |
| Proton pump inhibitors (A02BC) | Clostridium difficile infection | Intestinal anti-infectives (A07A) | 149 [109/40] | 2.73 (1.90,3.91) | 2.88 (2.00,4.13) |
| Corticosteroids (systemic use) (H02A) | Psychiatric disorders | Antipsychotics (N05A) | 2239 [1597/642] | 2.49 (2.27,2.73) | 2.50 (2.28,2.74) |
| Erythromycin (J01FA01) | Arrhythmia | Antiarrhythmics (C01B) | 61 [40/21] | 1.90 (1.12,3.23) | 2.39 (1.41,4.05) |
| Erythromycin (J01FA01) | Arrhythmia | Antithrombotic (B01AA B01AE B01AF) | 279 [175/104] | 1.68 (1.32,2.14) | 1.82 (1.43,2.32) |
| HMG CoA reductase inhibitors (C10AA) (statins) | Arrhythmia | Antiarrhythmics (C01B) | 262 [166/96] | 1.73 (1.34,2.22) | 1.78 (1.38,2.28) |
| Tricyclic antidepressant (N06AA) | Cognitive impairment | Anti-dementia (N06D) | 275 [174/101] | 1.72 (1.35,2.20) | 1.76 (1.38,2.25) |
| Amiodarone (C01BD01) | Hypothyroidism | Levothyroxine (H03AA01) | 237 [152/85] | 1.79 (1.37,2.33) | 1.74 (1.34,2.27) |
| Calcium channel blockers (C08) | Constipation | Laxative (A06A) | 2715 [1699/1016] | 1.67 (1.55,1.81) | 1.70 (1.57,1.84) |
| Dopaminergic agent (N04B) | Paradoxical agitation | Antipsychotics (N05A) | 483 [299/184] | 1.63 (1.35,1.95) | 1.70 (1.41,2.04) |
| HMG CoA reductase inhibitors (C10AA) (statins) | Arrhythmia | Antithrombotic (B01AA B01AE B01AF) | 1073 [676/397] | 1.70 (1.50,1.93) | 1.68 (1.49,1.91) |
| HMG CoA reductase inhibitors (C10AA) (statins) | Cognitive impairment | Anti-dementia (N06D) | 644 [410/234] | 1.75 (1.49,2.06) | 1.66 (1.41,1.95) |
| Prazosin (C02CA01) | Urinary incontinence | Oxybutynin (G04BD04) | 141 [89/52] | 1.71 (1.22,2.41) | 1.64 (1.16,2.30) |
| HMG CoA reductase inhibitors (C10AA) (statins) | Depression | Antidepressants (N06A) | 1813 [1112/701] | 1.59 (1.44,1.74) | 1.60 (1.45,1.76) |
| Metformin (A10BA02) | Diarrhea | Antidiarrheal (A07) | 257 [157/100] | 1.57 (1.22,2.02) | 1.55 (1.21,1.99) |
| Non-steroidal anti-inflammatory drugs (NSAIDs) (M01A) | Nausea/dizziness | Antiemetic (A03FA03 N05AD08 A03FA01 A04AD A04AA) | 3016 [1821/1195] | 1.52 (1.42,1.64) | 1.55 (1.44,1.66) |
| Non-steroidal anti-inflammatory drugs (NSAIDs) (M01A) | Heart failure | HF beta-blocker (C07AB07 C07AG02 C07AB02 C07AB12) | 1450 [867/583] | 1.49 (1.34,1.65) | 1.53 (1.38,1.70) |
| Anticholinergic (R03BB) | Urinary retention in BPH | Dutasteride Finasteride Tamsulosin Dutasteride/Tamsulosin Alfuzosin Silodosin (G04CB02 G04CB01 G04CA02 G04CA52 G04CA01 G04CA04) | 227 [134/93] | 1.44 (1.11,1.88) | 1.48 (1.13,1.92) |
| Beta blocking agents (C07) | Depression | TCA SSRI Other Antidepressant (N06AA N06AB N06AX) | 2628 [1565/1063] | 1.47 (1.36,1.59) | 1.46 (1.35,1.58) |
| Antihypertensives (C02 C03 C07 C08 C09) | Nausea/dizziness | Antiemetic (A03FA03 N05AD08 A03FA01 A04AD A04AA) | 2513 [1482/1031] | 1.44 (1.33,1.56) | 1.44 (1.33,1.56) |
| Benzodiazepine derivatives (N05BA N05CD) | Paradoxical agitation | Antipsychotics (N05A) | 4479 [2640/1839] | 1.44 (1.35,1.52) | 1.43 (1.35,1.52) |
| Tricyclic antidepressant (N06AA) | Constipation | Laxative (A06A) | 1791 [1048/743] | 1.41 (1.28,1.55) | 1.42 (1.29,1.56) |
| HMG CoA reductase inhibitors (C10AA) (statins) | Sleeplessness | Hypnotics and sedatives (N05C) | 1214 [710/504] | 1.41 (1.26,1.58) | 1.42 (1.26,1.59) |
| Opioids (N02A) | Nausea/dizziness | Antiemetic (A03FA03 N05AD08 A03FA01 A04AD A04AA) | 8675 [5077/3598] | 1.41 (1.35,1.47) | 1.42 (1.36,1.48) |
| Non-steroidal anti-inflammatory drugs (NSAIDs) (M01A) | GI symptoms | Anti-ulcer (A02B) | 2390 [1390/1000] | 1.39 (1.28,1.51) | 1.41 (1.30,1.53) |
| Non-steroidal anti-inflammatory drugs (NSAIDs) (M01A) | Edema peripheral | High-ceiling diuretics (C03C) | 2932 [1700/1232] | 1.38 (1.28,1.48) | 1.39 (1.29,1.49) |
| Antiadrenergic/Antimigraine (C02A N02CX) | Depression | TCA SSRI Other Antidepressant (N06AA N06AB N06AX) | 369 [209/160] | 1.31 (1.06,1.60) | 1.37 (1.11,1.68) |
| Opioids (N02A) | Depression | Antidepressants (N06A) | 6915 [3969/2946] | 1.35 (1.28,1.41) | 1.35 (1.29,1.42) |
| Dihydropyridines (C08CA) | Edema peripheral | High-ceiling diuretics (C03C) | 2345 [1338/1007] | 1.33 (1.22,1.44) | 1.33 (1.23,1.45) |
| Corticosteroids (systemic use) (H02A) | Insomnia | Benzodiazepine derivatives (N05BA N05CD) | 4506 [2533/1973] | 1.28 (1.21,1.36) | 1.31 (1.24,1.39) |
| Selective CCB (cardiac effects) (C08D C09BB10) | Depression | TCA SSRI Other Antidepressant (N06AA N06AB N06AX) | 288 [158/130] | 1.22 (0.96,1.53) | 1.28 (1.01,1.61) |
| Selective CCB (cardiac effects) (C08D C09BB10) | Edema peripheral | High-ceiling diuretics (C03C) | 524 [288/236] | 1.22 (1.03,1.45) | 1.27 (1.07,1.51) |
| Proton pump inhibitors (A02BC) | Bone loss | Bisphosphonates (M05BA) | 597 [337/260] | 1.30 (1.10,1.52) | 1.26 (1.07,1.49) |
| Direct anticoagulants (DOC) (B01AE07 B01AF01 B01AF02) | Depression | Antidepressants (N06A) | 2014 [1103/911] | 1.21 (1.11,1.32) | 1.24 (1.13,1.35) |
| Digoxin (C01AA05) | Nausea/dizziness | Antiemetic (A03FA03 N05AD08 A03FA01 A04AD A04AA) | 1443 [798/645] | 1.24 (1.12,1.37) | 1.23 (1.11,1.36) |
| Dihydropyridines (C08CA) | Depression | Antidepressants (N06A) | 1742 [948/794] | 1.19 (1.09,1.31) | 1.21 (1.10,1.33) |
| Cholinesterase inhibitor (N06DA) | Nausea/dizziness | Antiemetic (A03FA03 N05AD08 A03FA01 A04AD A04AA) | 1084 [577/507] | 1.14 (1.01,1.28) | 1.18 (1.05,1.33) |
| Cholinesterase inhibitor (N06DA) | Insomnia | Benzodiazepine derivatives (N05BA N05CD) | 1448 [763/685] | 1.11 (1.00,1.23) | 1.15 (1.04,1.28) |
| Antihypertensives (C02 C03 C07 C08 C09) | Nausea/dizziness | Prochlorperazine (A04AD) | 1069 [562/507] | 1.11 (0.98,1.25) | 1.13 (1.00,1.28) |
| ACE-inhibitors (C09A) | Depression | Antidepressants (N06A) | 1883 [990/893] | 1.11 (1.01,1.21) | 1.12 (1.02,1.22) |

| **Appendix 6:** Statistically significant prescribing cascades (n = 43) during the 18 months after LTCF transition (ordered by adjusted sequence ratio). Presented in Figure 2 of the main manuscript. | | | | | |
| --- | --- | --- | --- | --- | --- |
| **Index Medication** | **Potential ADR** | **Marker Medication** | **Incident users**  **[I-M/M-I]** | **Crude SR**  **(95%CI)** | **Adjusted SR (95%CI)** |
| Proton pump inhibitors (A02BC) | Clostridium difficile infection | Intestinal anti-infectives (A07A) | 89 [64/25] | 2.56 (1.61,4.06) | 3.40 (2.14,5.40) |
| HMG CoA reductase inhibitors (C10AA) (statins) | Skin soft tissue infection | Antibiotic (J01) | 4318 [3320/998] | 3.33 (3.10,3.57) | 3.20 (2.98,3.44) |
| ACE-inhibitors (C09A) | Cough | Antitussive (R05F R05D) | 66 [47/19] | 2.47 (1.45,4.21) | 3.02 (1.77,5.15) |
| HMG CoA reductase inhibitors (C10AA) (statins) | Confusion state | Antipsychotics (N05A) | 1383 [1032/351] | 2.94 (2.60,3.32) | 2.93 (2.59,3.31) |
| Digoxin (C01AA05) | Nausea/dizziness | Antiemetic (A03FA03 N05AD08 A03FA01 A04AD A04AA) | 2050 [1513/537] | 2.82 (2.55,3.11) | 2.81 (2.54,3.10) |
| Antihypertensives (C02 C03 C07 C08 C09) | Nausea/dizziness | Antiemetic (A03FA03 N05AD08 A03FA01 A04AD A04AA) | 4963 [3628/1335] | 2.72 (2.55,2.89) | 2.68 (2.52,2.86) |
| HMG CoA reductase inhibitors (C10AA) (statins) | Myalgia/myositis | Non-steroidal anti-inflammatory drugs (NSAIDs) (M01A) | 581 [426/155] | 2.75 (2.29,3.30) | 2.59 (2.16,3.11) |
| Cholinesterase inhibitor (N06DA) | Orthostatic hypotension | Prochlorperazine (A04AD) | 116 [78/38] | 2.05 (1.39,3.02) | 2.44 (1.65,3.59) |
| HMG CoA reductase inhibitors (C10AA) (statins) | Depression | Antidepressants (N06A) | 1433 [1001/432] | 2.32 (2.07,2.59) | 2.29 (2.05,2.56) |
| Amiodarone (C01BD01) | Hypothyroidism | Levothyroxine (H03AA01) | 91 [59/32] | 1.84 (1.20,2.84) | 2.26 (1.47,3.48) |
| HMG CoA reductase inhibitors (C10AA) (statins) | Arrhythmia | Antiarrhythmics (C01B) | 63 [43/20] | 2.15 (1.26,3.65) | 2.19 (1.29,3.72) |
| Metformin (A10BA02) | Diarrhea | Antidiarrheal (A07) | 163 [110/53] | 2.08 (1.50,2.88) | 2.14 (1.54,2.97) |
| Cholinesterase inhibitor (N06DA) | Nausea/dizziness | Antiemetic (A03FA03 N05AD08 A03FA01 A04AD A04AA) | 786 [539/247] | 2.18 (1.88,2.54) | 2.14 (1.84,2.49) |
| HMG CoA reductase inhibitors (C10AA) (statins) | Arrhythmia | Antithrombotic (B01AA B01AE B01AF) | 535 [357/178] | 2.01 (1.68,2.40) | 2.12 (1.77,2.54) |
| HMG CoA reductase inhibitors (C10AA) (statins) | Sleeplessness | Hypnotics and sedatives (N05C) | 1164 [789/375] | 2.10 (1.86,2.38) | 2.08 (1.84,2.36) |
| Dihydropyridines (C08CA) | Edema peripheral | High-ceiling diuretics (C03C) | 1945 [1298/647] | 2.01 (1.83,2.20) | 1.94 (1.76,2.13) |
| HMG CoA reductase inhibitors (C10AA) (statins) | Cognitive impairment | Anti-dementia (N06D) | 183 [121/62] | 1.95 (1.44,2.65) | 1.85 (1.36,2.52) |
| Selective CCB (cardiac effects) (C08D C09BB10) | Depression | TCA SSRI Other Antidepressant (N06AA N06AB N06AX) | 163 [104/59] | 1.76 (1.28,2.43) | 1.83 (1.33,2.51) |
| Beta blocking agents (C07) | Depression | TCA SSRI Other Antidepressant (N06AA N06AB N06AX) | 2343 [1534/809] | 1.90 (1.74,2.06) | 1.82 (1.67,1.98) |
| Antihypertensives (C02 C03 C07 C08 C09) | Nausea/dizziness | Prochlorperazine (A04AD) | 720 [463/257] | 1.80 (1.55,2.10) | 1.79 (1.54,2.08) |
| ACE-inhibitors (C09A) | Cough | Antibacterials (cough) (J01A J01B J01F J01G J01R) | 2573 [1642/931] | 1.76 (1.63,1.91) | 1.72 (1.59,1.86) |
| Cholinesterase inhibitor (N06DA) | Diarrhea | Antidiarrheal (A07) | 76 [48/28] | 1.71 (1.08,2.73) | 1.66 (1.04,2.65) |
| Opioids (N02A) | Nausea/dizziness | Antiemetic (A03FA03 N05AD08 A03FA01 A04AD A04AA) | 18944 [11816/7128] | 1.66 (1.61,1.71) | 1.66 (1.61,1.71) |
| Benzodiazepine derivatives (N05BA N05CD) | Paradoxical agitation | Antipsychotics (N05A) | 10724 [6490/4234] | 1.53 (1.47,1.59) | 1.53 (1.47,1.59) |
| ACE-inhibitors (C09A) | Arthritis | Non-steroidal anti-inflammatory drugs (NSAIDs) (M01A) | 695 [417/278] | 1.50 (1.29,1.75) | 1.51 (1.30,1.76) |
| HMG CoA reductase inhibitors (C10AA) (statins) | Urinary incontinence | Oxybutynin (G04BD04) | 141 [82/59] | 1.39 (0.99,1.94) | 1.44 (1.03,2.01) |
| Proton pump inhibitors (A02BC) | Pneumonia | Antibiotic (J01) | 10103 [5983/4120] | 1.45 (1.40,1.51) | 1.43 (1.38,1.49) |
| ACE-inhibitors (C09A) | Depression | Antidepressants (N06A) | 1722 [1011/711] | 1.42 (1.29,1.57) | 1.40 (1.27,1.54) |
| Antiepileptics (other) (N03AX) | Urinary tract infection (UTI) | Antibacterials (UTI) (J01C J01D J01E J01M J01X) | 1302 [772/530] | 1.46 (1.30,1.63) | 1.40 (1.25,1.56) |
| Dihydropyridines (C08CA) | Depression | Antidepressants (N06A) | 1551 [906/645] | 1.40 (1.27,1.55) | 1.39 (1.25,1.53) |
| Laxative (A06A) | Diarrhea | Antidiarrheal (A07) | 1604 [948/656] | 1.45 (1.31,1.60) | 1.39 (1.25,1.53) |
| Benzodiazepine derivatives (N05BA N05CD) | Nausea/dizziness | Prochlorperazine (A04AD) | 1589 [922/667] | 1.38 (1.25,1.53) | 1.36 (1.23,1.51) |
| Proton pump inhibitors (A02BC) | Bone loss | Denosumab (M05BX04) | 1889 [1077/812] | 1.33 (1.21,1.45) | 1.27 (1.16,1.40) |
| Non-steroidal anti-inflammatory drugs (NSAIDs) (M01A) | Nausea/dizziness | Antiemetic (A03FA03 N05AD08 A03FA01 A04AD A04AA) | 3500 [1950/1550] | 1.26 (1.18,1.34) | 1.27 (1.19,1.35) |
| Direct anticoagulants (DOC) (B01AE07 B01AF01 B01AF02) | Depression | Antidepressants (N06A) | 1524 [838/686] | 1.22 (1.10,1.35) | 1.23 (1.11,1.36) |
| Antiepileptics (N03A) | Rash topical | Corticosteroid (D07) | 2097 [1162/935] | 1.24 (1.14,1.35) | 1.21 (1.11,1.32) |
| Benzodiazepine derivatives (N05BA N05CD) | Cognitive impairment | Anti-dementia (N06D) | 1224 [668/556] | 1.20 (1.07,1.34) | 1.20 (1.07,1.34) |
| Antidepressants (N06A) | Parkinsonism | Anti-Parkinson drugs (N04) | 621 [331/290] | 1.14 (0.97,1.34) | 1.19 (1.02,1.40) |
| SSRI/SNRI (N06AX23 N06AX16 N06AX21 N06AB) | Insomnia | Antidepressant (sedating) (N06AX11 N06AX05 N06AA09 N06AA12 N06AA10 N06AX22) | 2908 [1556/1352] | 1.15 (1.07,1.24) | 1.17 (1.09,1.26) |
| Calcium channel blockers (CCB) (C08) | Constipation | Laxative (A06A) | 3156 [1703/1453] | 1.17 (1.09,1.26) | 1.16 (1.08,1.25) |
| Antiepileptics (N03A) | Nausea/dizziness | Antiemetic (A03FA03 N05AD08 A03FA01 A04AD A04AA) | 2747 [1458/1289] | 1.13 (1.05,1.22) | 1.14 (1.05,1.22) |
| ACE-inhibitors (C09A) | Cough | Adrenergics/inhalants (R03A) | 1668 [888/780] | 1.14 (1.03,1.25) | 1.13 (1.03,1.25) |
| Benzodiazepine derivatives (N05BA N05CD) | Edema peripheral | High-ceiling diuretics (C03C) | 8302 [4341/3961] | 1.10 (1.05,1.14) | 1.09 (1.05,1.14) |

| **Appendix 7:** Non-statistically significant prescribing cascades during the 18 months before and after LTCF transition (ordered by adjusted sequence ratio). | | | | | |
| --- | --- | --- | --- | --- | --- |
| **Index Medication** | **Potential ADR** | **Marker Medication** | **Incident users**  **[I-M/M-I]** | **Crude SR**  **(95%CI)** | **Adjusted SR (95%CI)** |
| **Before transition to LTCF** | | | | | |
| Prazosin (C02CA01) | Orthostatic hypotension | Prochlorperazine (A04AD) | 147 [81/66] | 1.23 (0.89,1.70) | 1.32 (0.96,1.83) |
| Brinzolamide (S01EC04) | Edema peripheral | High-ceiling diuretics (C03C) | 128 [70/58] | 1.21 (0.85,1.71) | 1.22 (0.86,1.72) |
| Antidepressants (N06A) | Parkinsonism | Anti-Parkinson drugs (N04) | 823 [441/382] | 1.15 (1.01,1.32) | 1.14 (0.99,1.31) |
| Urinary frequency incontinence (G04BD10 G04BD04 G04BD08 G04BD07) | Cognitive impairment | Anti-dementia (N06D) | 307 [163/144] | 1.13 (0.90,1.42) | 1.14 (0.91,1.42) |
| Inhaled corticosteroids (R03BA) | Peptic ulcer | Proton pump inhibitors (A02BC) | 323 [171/152] | 1.13 (0.90,1.40) | 1.13 (0.91,1.41) |
| Non-steroidal anti-inflammatory drugs (NSAIDs) (M01A) | Nausea/dizziness | Prochlorperazine (A04AD) | 1276 [669/607] | 1.10 (0.99,1.23) | 1.11 (1.00,1.24) |
| ACE-inhibitors (C09A) | Cough | Antihistamines (systemic use) (R06A) | 57 [30/27] | 1.11 (0.66,1.87) | 1.11 (0.66,1.87) |
| Coxibs (M01AH) | Hypertension | Antihypertensives (C02 C03 C07 C08 C09) | 553 [282/271] | 1.04 (0.88,1.23) | 1.11 (0.94,1.31) |
| Inhaled corticosteroids (R03BA) | Edema peripheral | High-ceiling diuretics (C03C) | 538 [270/268] | 1.01 (0.85,1.19) | 1.09 (0.92,1.29) |
| High-ceiling diuretics (C03C) | Diuresis excessive | Benign prostatic hypertrophy (G04C) | 818 [423/395] | 1.07 (0.93,1.23) | 1.08 (0.94,1.23) |
| SSRI/SNRI (N06AX23 N06AX16 N06AX21 N06AB) | Insomnia | Antidepressant (sedating) (N06AX11 N06AX05 N06AA09 N06AA12 N06AA10 N06AX22) | 2295 [1175/1120] | 1.05 (0.97,1.14) | 1.07 (0.99,1.16) |
| Latanoprost (S01EE01) | Edema peripheral | High-ceiling diuretics (C03C) | 424 [215/209] | 1.03 (0.85,1.24) | 1.06 (0.88,1.28) |
| Corticosteroids (systemic use) (H02A) | Diabetes | Blood lowering medications (A10B) | 794 [390/404] | 0.97 (0.84,1.11) | 1.04 (0.91,1.20) |
| Cholinesterase inhibitor (N06DA) | Diarrhea | Antidiarrheal (A07) | 254 [129/125] | 1.03 (0.81,1.32) | 1.03 (0.80,1.31) |
| Metoclopramide (A03FA01) | Parkinsonism | Anti-Parkinson drugs (N04) | 381 [186/195] | 0.95 (0.78,1.17) | 0.99 (0.81,1.21) |
| HMG CoA reductase inhibitors (C10AA) (statins) | Urinary incontinence | Oxybutynin (G04BD04) | 381 [189/192] | 0.98 (0.81,1.20) | 0.97 (0.79,1.18) |
| Lithium (N06AX) | Hypothyroidism | Levothyroxine (H03AA01) | 47 [22/25] | 0.88 (0.50,1.56) | 0.95 (0.54,1.69) |
| Non-steroidal anti-inflammatory drugs (NSAIDs) (M01A) | Hypertension | Antihypertensives (C02 C03 C07 C08 C09) | 1684 [812/872] | 0.93 (0.85,1.02) | 0.95 (0.86,1.04) |
| Proton pump inhibitors (A02BC) | Bone loss | Denosumab (M05BX04) | 2154 [1049/1105] | 0.95 (0.87,1.03) | 0.94 (0.87,1.03) |
| Low-ceiling diuretics (C03B C03A) | Gout | Anti-gout medication (M04A) | 386 [185/201] | 0.92 (0.75,1.12) | 0.93 (0.76,1.14) |
| Tetracyclines (J01A) | Arthritis | Non-steroidal anti-inflammatory drugs (NSAIDs) (M01A) | 1733 [828/905] | 0.91 (0.83,1.01) | 0.93 (0.84,1.02) |
| For urinary frequency and incontinence (G04BD) | Hypertension | Antihypertensives (C02 C03 C07 C08 C09) | 549 [258/291] | 0.89 (0.75,1.05) | 0.91 (0.77,1.08) |
| High-ceiling diuretics (C03C) | Urinary incontinence | Oxybutynin (G04BD04) | 1124 [529/595] | 0.89 (0.79,1.00) | 0.90 (0.80,1.01) |
| Anti-dementia (N06D) | Urinary incontinence | Oxybutynin (G04BD04) | 307 [144/163] | 0.88 (0.71,1.11) | 0.89 (0.71,1.11) |
| Lithium (N06AX) | Tremor | Propranolol (C07AA05) | 139 [64/75] | 0.85 (0.61,1.19) | 0.88 (0.63,1.23) |
| Cholinesterase inhibitor (N06DA) | Urinary incontinence | Oxybutynin (G04BD04) | 293 [137/156] | 0.88 (0.70,1.10) | 0.87 (0.69,1.09) |
| Ranitidine (A02BA02) | Heart failure | HF beta-blocker (C07AB07 C07AG02 C07AB02 C07AB12) | 403 [183/220] | 0.83 (0.68,1.01) | 0.84 (0.69,1.02) |
| Anticholinergic (R03BB) | Urinary retention | Prazosin (C02CA01) | 144 [65/79] | 0.82 (0.59,1.14) | 0.82 (0.59,1.13) |
| Memantine (N06DX01) | Seizures | Antiepileptics (N03) | 92 [45/47] | 0.96 (0.64,1.44) | 0.81 (0.54,1.22) |
| Carbamazepine (N03AF01) | Hypothyroidism | Levothyroxine (H03AA01) | 58 [25/33] | 0.76 (0.45,1.27) | 0.73 (0.43,1.22) |
| **After transition to LTCF** | | | | | |
| ACE-inhibitors (C09A) | Cough | Antihistamines (systemic use) (R06A) | 38 [24/14] | 1.71 (0.89,3.31) | 1.88 (0.97,3.63) |
| Benign Prostatic Hyperplasia (G04CB02 G04CB01 G04CA02 G04CA52 G04CA01 G04CA04) | Dizziness | Prochlorperazine (A04AD) | 107 [62/45] | 1.38 (0.94,2.02) | 1.40 (0.96,2.06) |
| Tricyclic antidepressant (N06AA) | Cognitive impairment | Anti-dementia (N06D) | 86 [47/39] | 1.21 (0.79,1.84) | 1.36 (0.89,2.07) |
| Tricyclic antidepressant (N06AA) | Cognitive impairment | Anti-dementia (N06D) | 86 [47/39] | 1.21 (0.79,1.84) | 1.36 (0.89,2.07) |
| Prazosin (C02CA01) | Urinary incontinence | Oxybutynin (G04BD04) | 58 [33/25] | 1.32 (0.79,2.22) | 1.34 (0.80,2.26) |
| Prazosin (C02CA01) | Orthostatic hypotension | Prochlorperazine (A04AD) | 73 [39/34] | 1.15 (0.72,1.82) | 1.28 (0.81,2.02) |
| Cholinesterase inhibitor (N06DA) | Urinary incontinence | Oxybutynin (G04BD04) | 61 [32/29] | 1.10 (0.67,1.82) | 1.23 (0.74,2.03) |
| Latanoprost (S01EE01) | Edema peripheral | High-ceiling diuretics (C03C) | 385 [212/173] | 1.23 (1.00,1.50) | 1.21 (0.99,1.48) |
| Cholinesterase inhibitor (N06DA) | Peptic ulcer | Proton pump inhibitors (A02BC) | 351 [184/167] | 1.10 (0.89,1.36) | 1.15 (0.93,1.41) |
| Dopaminergic agent (N04B) | Paradoxical agitation | Antipsychotics (N05A) | 580 [301/279] | 1.08 (0.92,1.27) | 1.12 (0.95,1.31) |
| High-ceiling diuretics (C03C) | Diuresis excessive | Benign prostatic hypertrophy (G04C) | 605 [325/280] | 1.16 (0.99,1.36) | 1.11 (0.95,1.31) |
| Anti-dementia (N06D) | Urinary incontinence | Oxybutynin (G04BD04) | 70 [36/34] | 1.06 (0.66,1.69) | 1.11 (0.69,1.77) |
| Selective CCB (cardiac effects) (C08D C09BB10) | Edema peripheral | High-ceiling diuretics (C03C) | 226 [120/106] | 1.13 (0.87,1.47) | 1.10 (0.85,1.43) |
| Gabapentinoid (N02BF) | Nausea/dizziness | Prochlorperazine (A04AD) | 556 [295/261] | 1.13 (0.96,1.34) | 1.10 (0.93,1.30) |
| Antipsychotics (N05A) | Parkinsonism/Extrapyramidal symptoms | Anti-Parkinson drugs (N04) | 648 [334/314] | 1.06 (0.91,1.24) | 1.07 (0.92,1.25) |
| Dipeptidyl peptidase 4 (DPP-4) inhibitors (A10BH) | Arthritis | Non-steroidal anti-inflammatory drugs (NSAIDs) (M01A) | 204 [107/97] | 1.10 (0.84,1.45) | 1.06 (0.81,1.40) |
| Tricyclic antidepressant (N06AA) | Shortness of beath | Adrenergics/inhalants (R03A) | 845 [430/415] | 1.04 (0.91,1.19) | 1.06 (0.92,1.21) |
| Proton pump inhibitors (A02BC) | Bone loss | Bisphosphonates (M05BA) | 303 [152/151] | 1.01 (0.80,1.26) | 1.04 (0.83,1.31) |
| Opioids (N02A) | Nausea/dizziness | Prochlorperazine (A04AD) | 2462 [1251/1211] | 1.03 (0.95,1.12) | 1.03 (0.95,1.12) |
| Non-steroidal anti-inflammatory drugs (NSAIDs) (M01A) | Nausea/dizziness | Prochlorperazine (A04AD) | 594 [299/295] | 1.01 (0.86,1.19) | 1.03 (0.88,1.21) |
| Lithium (N06AX) | Parkinsonism | Anti-Parkinson drugs (N04) | 435 [216/219] | 0.99 (0.82,1.19) | 1.03 (0.85,1.24) |
| Antiadrenergic/Antimigraine (C02A N02CX) | Depression | TCA SSRI Other Antidepressant (N06AA N06AB N06AX) | 204 [100/104] | 0.96 (0.73,1.27) | 1.02 (0.77,1.34) |
| Corticosteroids (systemic use) (H02A) | Psychiatric disorders | Antipsychotics (N05A) | 3900 [1947/1953] | 1.00 (0.94,1.06) | 1.01 (0.95,1.08) |
| Bisphosphonates (M05BA) | Gastrointestinal disorders | Medications for acid disorders (A02A A02BA A02BC A02BX) | 306 [156/150] | 1.04 (0.83,1.30) | 1.01 (0.81,1.26) |
| Memantine (N06DX01) | Seizures | Antiepileptics (N03) | 266 [126/140] | 0.90 (0.71,1.14) | 0.97 (0.77,1.24) |
| Urinary frequency incontinence (G04BD10 G04BD04 G04BD08 G04BD07) | Cognitive impairment | Anti-dementia (N06D) | 70 [34/36] | 0.94 (0.59,1.51) | 0.97 (0.61,1.55) |
| Bisphosphonates (M05BA) | Peptic ulcer | Proton pump inhibitors (A02BC) | 303 [151/152] | 0.99 (0.79,1.24) | 0.97 (0.78,1.22) |
| Cholinesterase inhibitor (N06DA) | Insomnia | Benzodiazepine derivatives (N05BA N05CD) | 962 [476/486] | 0.98 (0.86,1.11) | 0.97 (0.86,1.10) |
| Brinzolamide (S01EC04) | Edema peripheral | High-ceiling diuretics (C03C) | 96 [50/46] | 1.09 (0.73,1.62) | 0.97 (0.65,1.45) |
| Non-steroidal anti-inflammatory drugs (NSAIDs) (M01A) | GI symptoms | Anti-ulcer (A02B) | 1737 [841/896] | 0.94 (0.85,1.03) | 0.96 (0.88,1.06) |
| Gabapentinoid (N02BF) | Edema peripheral | High-ceiling diuretics (C03C) | 2385 [1153/1232] | 0.94 (0.86,1.01) | 0.94 (0.87,1.02) |
| Low-ceiling diuretics (C03B C03A) | Gout | Anti-gout medication (M04A) | 168 [81/87] | 0.93 (0.69,1.26) | 0.94 (0.70,1.28) |
| Anticholinergic (R03BB) | Urinary retention in BPH | Dutasteride, Finasteride, Tamsulosin, Dutasteride/Tamsulosin, Alfuzosin, Silodosin (G04CB02, G04CB01, G04CA02, G04CA52, G04CA01, G04CA04) | 201 [94/107] | 0.88 (0.67,1.16) | 0.92 (0.70,1.21) |
| High-ceiling diuretics (C03C) | Urinary incontinence | Oxybutynin (G04BD04) | 807 [375/432] | 0.87 (0.76,1.00) | 0.90 (0.79,1.04) |
| Lithium (N06AX) | Tremor | Propranolol (C07AA05) | 113 [55/58] | 0.95 (0.66,1.37) | 0.86 (0.59,1.24) |
| Tricyclic antidepressant (N06AA) | Orthostatic hypotension | Prochlorperazine (A04AD) | 320 [149/171] | 0.87 (0.70,1.09) | 0.85 (0.69,1.06) |
| Antipsychotics (N05A) | Urinary retention in BPH | Dutasteride, Finasteride, Tamsulosin, Dutasteride/Tamsulosin, Alfuzosin, Silodosin (G04CB02, G04CB01, G04CA02, G04CA52, G04CA01, G04CA04) | 493 [230/263] | 0.87 (0.73,1.04) | 0.84 (0.71,1.01) |
| Carbamazepine (N03AF01) | Hypothyroidism | Levothyroxine (H03AA01) | 42 [17/25] | 0.68 (0.37,1.26) | 0.57 (0.31,1.06) |
| Erythromycin (J01FA01) | Arrhythmia | Antiarrhythmics (C01B) | 34 [12/22] | 0.55 (0.27,1.10) | 0.55 (0.27,1.10) |

| **Appendix 8:** Summary of statistically significant prescribing cascades occurring before and after entry to LTCF. | | |
| --- | --- | --- |
| **Index medication (ATC)** | **Potential ADR** | **Marker medication (ATC)** |
| **Cascades occurring only after LTCF (n = 20)** | | |
| ACE-inhibitors (C09A) | Cough | Antitussive (R05F R05D) |
| ACE-inhibitors (C09A) | Arthritis | Non-steroidal anti-inflammatory drugs (NSAIDs) (M01A) |
| ACE-inhibitors (C09A) | Cough | Antibacterials (cough) (J01A J01B J01F J01G J01R) |
| ACE-inhibitors (C09A) | Cough | Adrenergics/inhalants (R03A) |
| Antidepressants (N06A) | Parkinsonism | Anti-Parkinson drugs (N04) |
| Antiepileptics (N03A) | Nausea/dizziness | Antiemetic (A03FA03 N05AD08 A03FA01 A04AD A04AA) |
| Antiepileptics (N03A) | Rash topical | Corticosteroid (D07) |
| Antiepileptics (other) (N03AX) | Urinary tract infection (UTI) | Antibacterials (UTI) (J01C J01D J01E J01M J01X) |
| Benzodiazepine derivatives (N05BA N05CD) | Cognitive impairment | Anti-dementia (N06D) |
| Benzodiazepine derivatives (N05BA N05CD) | Nausea/dizziness | Prochlorperazine (A04AD) |
| Benzodiazepine derivatives (N05BA N05CD) | Edema peripheral | High-ceiling diuretics (C03C) |
| Cholinesterase inhibitor (N06DA) | Orthostatic hypotension | Prochlorperazine (A04AD) |
| Cholinesterase inhibitor (N06DA) | Diarrhea | Antidiarrheal (A07) |
| HMG CoA reductase inhibitors (C10AA) (statins) | Skin soft tissue infection | Antibiotic (J01) |
| HMG CoA reductase inhibitors (C10AA) (statins) | Myalgia/myositis | Non-steroidal anti-inflammatory drugs (NSAIDs) (M01A) |
| HMG CoA reductase inhibitors (C10AA) (statins) | Urinary incontinence | Oxybutynin (G04BD04) |
| Laxative (A06A) | Diarrhea | Antidiarrheal (A07) |
| Proton pump inhibitors (A02BC) | Bone loss | Denosumab (M05BX04) |
| Proton pump inhibitors (A02BC) | Pneumonia | Antibiotic (J01) |
| SSRI/SNRI (N06AX23 N06AX16 N06AX21 N06AB) | Insomnia | Antidepressant (sedating) (N06AX11 N06AX05 N06AA09 N06AA12 N06AA10 N06AX22) |
| **Cascades occurring only before LTCF (n = 17)** | | |
| Antiadrenergic/Antimigraine (C02A N02CX) | Depression | TCA SSRI Other Antidepressant (N06AA N06AB N06AX) |
| Anticholinergic (R03BB) | Urinary retention in BPH | Dutasteride, Finasteride, Tamsulosin, Dutasteride/Tamsulosin, Alfuzosin, Silodosin (G04CB02, G04CB01, G04CA02, G04CA52, G04CA01, G04CA04) |
| Non-steroidal anti-inflammatory drugs (NSAIDs) (M01A) | Edema peripheral | High-ceiling diuretics (C03C) |
| Non-steroidal anti-inflammatory drugs (NSAIDs) (M01A) | GI symptoms | Anti-ulcer (A02B) |
| Non-steroidal anti-inflammatory drugs (NSAIDs) (M01A) | Heart failure | HF beta-blocker (C07AB07 C07AG02 C07AB02 C07AB12) |
| Cholinesterase inhibitor (N06DA) | Insomnia | Benzodiazepine derivatives (N05BA N05CD) |
| Corticosteroids (systemic use) (H02A) | Insomnia | Benzodiazepine derivatives (N05BA N05CD) |
| Corticosteroids (systemic use) (H02A) | Psychiatric disorders | Antipsychotics (N05A) |
| Dopaminergic agent (N04B) | Paradoxical agitation | Antipsychotics (N05A) |
| Erythromycin (J01FA01) | Arrhythmia | Antiarrhythmics (C01B) |
| Erythromycin (J01FA01) | Arrhythmia | Antithrombotic (B01AA B01AE B01AF) |
| Opioids (N02A) | Depression | Antidepressants (N06A) |
| Prazosin (C02CA01) | Urinary incontinence | Oxybutynin (G04BD04) |
| Proton pump inhibitors (A02BC) | Bone loss | Bisphosphonates (M05BA) |
| Selective CCB (cardiac effects) (C08D C09BB10) | Edema peripheral | High-ceiling diuretics (C03C) |
| Tricyclic antidepressant (N06AA) | Constipation | Laxative (A06A) |
| Tricyclic antidepressant (N06AA) | Cognitive impairment | Anti-dementia (N06D) |
| **Cascades present in both periods (n = 23)** | | |
| ACE-inhibitors (C09A) | Depression | Antidepressants (N06A) |
| Amiodarone (C01BD01) | Hypothyroidism | Levothyroxine (H03AA01) |
| Antihypertensives (C02 C03 C07 C08 C09) | Nausea/dizziness | Antiemetic (A03FA03 N05AD08 A03FA01 A04AD A04AA) |
| Antihypertensives (C02 C03 C07 C08 C09) | Nausea/dizziness | Prochlorperazine (A04AD) |
| Non-steroidal anti-inflammatory drugs (NSAIDs) (M01A) | Nausea/dizziness | Antiemetic (A03FA03 N05AD08 A03FA01 A04AD A04AA) |
| Benzodiazepine derivatives (N05BA N05CD) | Paradoxical agitation | Antipsychotics (N05A) |
| Beta blocking agents (C07) | Depression | TCA SSRI Other Antidepressant (N06AA N06AB N06AX) |
| Calcium channel blockers (CCB) (C08) | Constipation | Laxative (A06A) |
| Cholinesterase inhibitor (N06DA) | Nausea/dizziness | Antiemetic (A03FA03 N05AD08 A03FA01 A04AD A04AA) |
| Digoxin (C01AA05) | Nausea/dizziness | Antiemetic (A03FA03 N05AD08 A03FA01 A04AD A04AA) |
| Dihydropyridines (C08CA) | Depression | Antidepressants (N06A) |
| Dihydropyridines (C08CA) | Edema peripheral | High-ceiling diuretics (C03C) |
| Direct anticoagulants (DOC) (B01AE07 B01AF01 B01AF02) | Depression | Antidepressants (N06A) |
| HMG CoA reductase inhibitors (C10AA) (statins) | Arrhythmia | Antiarrhythmics (C01B) |
| HMG CoA reductase inhibitors (C10AA) (statins) | Arrhythmia | Antithrombotic (B01AA B01AE B01AF) |
| HMG CoA reductase inhibitors (C10AA) (statins) | Cognitive impairment | Anti-dementia (N06D) |
| HMG CoA reductase inhibitors (C10AA) (statins) | Depression | Antidepressants (N06A) |
| HMG CoA reductase inhibitors (C10AA) (statins) | Sleeplessness | Hypnotics and sedatives (N05C) |
| HMG CoA reductase inhibitors (C10AA) (statins) | Confusion state | Antipsychotics (N05A) |
| Metformin (A10BA02) | Diarrhea | Antidiarrheal (A07) |
| Opioids (N02A) | Nausea/dizziness | Antiemetic (A03FA03 N05AD08 A03FA01 A04AD A04AA) |
| Proton pump inhibitors (A02BC) | Clostridium difficile infection | Intestinal anti-infectives (A07A) |
| Selective CCB (cardiac effects) (C08D C09BB10) | Depression | TCA SSRI Other Antidepressant (N06AA N06AB N06AX) |

| **Appendix 9:** Statistically significant prescribing cascades during the 18 months before entering LTCF, stratified by dementia status (ordered by adjusted sequence ratio). | | | | | |
| --- | --- | --- | --- | --- | --- |
| **Index Medication** | **Potential ADR** | **Marker Medication** | **Incident users**  **[I-M/M-I]** | **Crude SR**  **(95%CI)** | **Adjusted SR**  **(95%CI)** |
| **Among those living with dementia (n = 30 cascades)** | | | | | |
| Prazosin (C02CA01) | Orthostatic hypotension | Prochlorperazine (A04AD) | 32 [21/11] | 1.91 (0.92,3.96) | 3.47 (1.67,7.20) |
| HMG CoA reductase inhibitors (C10AA) (statins) | Confusion state | Antipsychotics (N05A) | 645 [486/159] | 3.06 (2.56,3.66) | 2.96 (2.47,3.54) |
| Prazosin (C02CA01) | Urinary incontinence | Oxybutynin (G04BD04) | 37 [26/11] | 2.36 (1.17,4.78) | 2.68 (1.32,5.42) |
| Corticosteroids (systemic use) (H02A) | Psychiatric disorders | Antipsychotics (N05A) | 1195 [834/361] | 2.31 (2.04,2.61) | 2.30 (2.03,2.60) |
| Metformin (A10BA02) | Diarrhea | Antidiarrheal (A07) | 64 [41/23] | 1.78 (1.07,2.97) | 2.28 (1.37,3.79) |
| Anticholinergic (R03BB) | Urinary retention in BPH | Dutasteride Finasteride Tamsulosin Dutasteride/Tamsulosin Alfuzosin Silodosin (G04CB02 G04CB01 G04CA02 G04CA52 G04CA01 G04CA04) | 48 [30/18] | 1.67 (0.93,2.99) | 2.27 (1.27,4.08) |
| HMG CoA reductase inhibitors (C10AA) (statins) | Sleeplessness | Hypnotics and sedatives (N05C) | 287 [187/100] | 1.87 (1.47,2.38) | 1.89 (1.48,2.41) |
| HMG CoA reductase inhibitors (C10AA) (statins) | Arrhythmia | Antithrombotic (B01AA B01AE B01AF) | 190 [121/69] | 1.75 (1.30,2.36) | 1.78 (1.32,2.39) |
| Tricyclic antidepressant (N06AA) | Cognitive impairment | Anti-dementia (N06D) | 264 [165/99] | 1.67 (1.30,2.14) | 1.72 (1.34,2.20) |
| Dopaminergic agent (N04B) | Paradoxical agitation | Antipsychotics (N05A) | 285 [177/108] | 1.64 (1.29,2.08) | 1.70 (1.34,2.16) |
| HMG CoA reductase inhibitors (C10AA) (statins) | Cognitive impairment | Anti-dementia (N06D) | 605 [385/220] | 1.75 (1.48,2.07) | 1.67 (1.41,1.97) |
| Tricyclic antidepressant (N06AA) | Constipation | Laxative (A06A) | 322 [197/125] | 1.58 (1.26,1.97) | 1.63 (1.30,2.04) |
| Beta blocking agents (C07) | Depression | TCA SSRI Other Antidepressant (N06AA N06AB N06AX) | 605 [370/235] | 1.57 (1.34,1.85) | 1.54 (1.31,1.81) |
| Antihypertensives (C02 C03 C07 C08 C09) | Nausea/dizziness | Antiemetic (A03FA03 N05AD08 A03FA01 A04AD A04AA) | 451 [271/180] | 1.51 (1.25,1.82) | 1.54 (1.27,1.86) |
| Calcium channel blockers (CCB) (C08) | Constipation | Laxative (A06A) | 569 [346/223] | 1.55 (1.31,1.84) | 1.51 (1.28,1.79) |
| Corticosteroids (systemic use) (H02A) | Insomnia | Benzodiazepine derivatives (N05BA N05CD) | 927 [551/376] | 1.47 (1.29,1.67) | 1.49 (1.31,1.70) |
| Non-steroidal anti-inflammatory drugs (NSAIDs) (M01A) | Heart failure | HF beta-blocker (C07AB07 C07AG02 C07AB02 C07AB12) | 267 [159/108] | 1.47 (1.15,1.88) | 1.48 (1.16,1.89) |
| HMG CoA reductase inhibitors (C10AA) (statins) | Depression | Antidepressants (N06A) | 506 [301/205] | 1.47 (1.23,1.75) | 1.47 (1.23,1.76) |
| Non-steroidal anti-inflammatory drugs (NSAIDs) (M01A) | Nausea/dizziness | Antiemetic (A03FA03 N05AD08 A03FA01 A04AD A04AA) | 545 [323/222] | 1.45 (1.23,1.73) | 1.46 (1.23,1.73) |
| Antidepressants (N06A) | Parkinsonism | Anti-Parkinson drugs (N04) | 216 [129/87] | 1.48 (1.13,1.95) | 1.40 (1.07,1.84) |
| Dihydropyridines (C08CA) | Edema peripheral | High-ceiling diuretics (C03C) | 368 [220/148] | 1.49 (1.21,1.83) | 1.40 (1.13,1.72) |
| Non-steroidal anti-inflammatory drugs (NSAIDs) (M01A) | GI symptoms | Anti-ulcer (A02B) | 562 [315/247] | 1.28 (1.08,1.51) | 1.34 (1.13,1.58) |
| Non-steroidal anti-inflammatory drugs (NSAIDs) (M01A) | Edema peripheral | High-ceiling diuretics (C03C) | 477 [268/209] | 1.28 (1.07,1.54) | 1.31 (1.09,1.57) |
| Benzodiazepine derivatives (N05BA N05CD) | Paradoxical agitation | Antipsychotics (N05A) | 3077 [1734/1343] | 1.29 (1.20,1.39) | 1.28 (1.19,1.38) |
| Direct anticoagulants (DOC) (B01AE07 B01AF01 B01AF02) | Depression | Antidepressants (N06A) | 443 [239/204] | 1.17 (0.97,1.41) | 1.24 (1.02,1.49) |
| Opioids (N02A) | Nausea/dizziness | Antiemetic (A03FA03 N05AD08 A03FA01 A04AD A04AA) | 1391 [750/641] | 1.17 (1.05,1.30) | 1.18 (1.06,1.31) |
| SSRI/SNRI (N06AX23 N06AX16 N06AX21 N06AB) | Insomnia | Antidepressant (sedating) (N06AX11 N06AX05 N06AA09 N06AA12 N06AA10 N06AX22) | 685 [376/309] | 1.22 (1.05,1.41) | 1.17 (1.01,1.36) |
| Cholinesterase inhibitor (N06DA) | Insomnia | Benzodiazepine derivatives (N05BA N05CD) | 1352 [714/638] | 1.12 (1.01,1.25) | 1.16 (1.04,1.29) |
| Cholinesterase inhibitor (N06DA) | Nausea/dizziness | Antiemetic (A03FA03 N05AD08 A03FA01 A04AD A04AA) | 1006 [532/474] | 1.12 (0.99,1.27) | 1.15 (1.02,1.31) |
| Opioids (N02A) | Depression | Antidepressants (N06A) | 1567 [824/743] | 1.11 (1.00,1.22) | 1.11 (1.00,1.22) |
| **Among those not living with dementia (n = 35 cascades)** | | | | | |
| HMG CoA reductase inhibitors (C10AA) (statins) | Confusion state | Antipsychotics (N05A) | 427 [316/111] | 2.85 (2.29,3.53) | 2.96 (2.39,3.68) |
| Corticosteroids (systemic use) (H02A) | Psychiatric disorders | Antipsychotics (N05A) | 1044 [763/281] | 2.72 (2.37,3.11) | 2.78 (2.42,3.18) |
| Proton pump inhibitors (A02BC) | Clostridium difficile infection | Intestinal anti-infectives (A07A) | 128 [92/36] | 2.56 (1.74,3.76) | 2.64 (1.80,3.88) |
| Erythromycin (J01FA01) | Arrhythmia | Antiarrhythmics (C01B) | 55 [36/19] | 1.89 (1.09,3.30) | 2.61 (1.50,4.55) |
| HMG CoA reductase inhibitors (C10AA) (statins) | Arrhythmia | Antiarrhythmics (C01B) | 221 [144/77] | 1.87 (1.42,2.47) | 1.97 (1.50,2.60) |
| Benzodiazepine derivatives (N05BA N05CD) | Paradoxical agitation | Antipsychotics (N05A) | 1402 [906/496] | 1.83 (1.64,2.04) | 1.88 (1.68,2.09) |
| Erythromycin (J01FA01) | Arrhythmia | Antithrombotic (B01AA B01AE B01AF) | 227 [144/83] | 1.73 (1.32,2.27) | 1.86 (1.42,2.44) |
| Cholinesterase inhibitor (N06DA) | Nausea/dizziness | Antiemetic (A03FA03 N05AD08 A03FA01 A04AD A04AA) | 78 [45/33] | 1.36 (0.87,2.14) | 1.74 (1.11,2.73) |
| Calcium channel blockers (CCB) (C08) | Constipation | Laxative (A06A) | 2146 [1353/793] | 1.71 (1.56,1.86) | 1.74 (1.59,1.90) |
| Amiodarone (C01BD01) | Hypothyroidism | Levothyroxine (H03AA01) | 208 [133/75] | 1.77 (1.34,2.35) | 1.69 (1.27,2.24) |
| HMG CoA reductase inhibitors (C10AA) (statins) | Depression | Antidepressants (N06A) | 1307 [811/496] | 1.64 (1.46,1.83) | 1.66 (1.49,1.86) |
| HMG CoA reductase inhibitors (C10AA) (statins) | Arrhythmia | Antithrombotic (B01AA B01AE B01AF) | 883 [555/328] | 1.69 (1.48,1.94) | 1.66 (1.45,1.90) |
| Dopaminergic agent (N04B) | Paradoxical agitation | Antipsychotics (N05A) | 198 [122/76] | 1.61 (1.21,2.14) | 1.63 (1.22,2.17) |
| Prazosin (C02CA01) | Urinary incontinence | Oxybutynin (G04BD04) | 104 [63/41] | 1.54 (1.04,2.28) | 1.61 (1.08,2.38) |
| Non-steroidal anti-inflammatory drugs (NSAIDs) (M01A) | Nausea/dizziness | Antiemetic (A03FA03 N05AD08 A03FA01 A04AD A04AA) | 2471 [1498/973] | 1.54 (1.42,1.67) | 1.57 (1.44,1.70) |
| Non-steroidal anti-inflammatory drugs (NSAIDs) (M01A) | Heart failure | HF beta-blocker (C07AB07 C07AG02 C07AB02 C07AB12) | 1183 [708/475] | 1.49 (1.33,1.67) | 1.52 (1.36,1.71) |
| Antiadrenergic/Antimigraine (C02A N02CX) | Depression | TCA SSRI Other Antidepressant (N06AA N06AB N06AX) | 293 [172/121] | 1.42 (1.13,1.79) | 1.47 (1.17,1.86) |
| Opioids (N02A) | Nausea/dizziness | Antiemetic (A03FA03 N05AD08 A03FA01 A04AD A04AA) | 7284 [4327/2957] | 1.46 (1.40,1.53) | 1.47 (1.40,1.54) |
| Beta blocking agents (C07) | Depression | TCA SSRI Other Antidepressant (N06AA N06AB N06AX) | 2023 [1195/828] | 1.44 (1.32,1.58) | 1.44 (1.32,1.58) |
| Anticholinergic (R03BB) | Urinary retention in BPH | Dutasteride Finasteride Tamsulosin Dutasteride/Tamsulosin Alfuzosin Silodosin (G04CB02 G04CB01 G04CA02 G04CA52 G04CA01 G04CA04) | 179 [104/75] | 1.39 (1.03,1.87) | 1.44 (1.07,1.94) |
| Non-steroidal anti-inflammatory drugs (NSAIDs) (M01A) | GI symptoms | Anti-ulcer (A02B) | 1828 [1075/753] | 1.43 (1.30,1.57) | 1.44 (1.31,1.58) |
| Opioids (N02A) | Depression | Antidepressants (N06A) | 5348 [3145/2203] | 1.43 (1.35,1.51) | 1.43 (1.35,1.51) |
| Antihypertensives (C02 C03 C07 C08 C09) | Nausea/dizziness | Antiemetic (A03FA03 N05AD08 A03FA01 A04AD A04AA) | 2062 [1211/851] | 1.42 (1.30,1.55) | 1.42 (1.30,1.55) |
| Non-steroidal anti-inflammatory drugs (NSAIDs) (M01A) | Edema peripheral | High-ceiling diuretics (C03C) | 2455 [1432/1023] | 1.40 (1.29,1.52) | 1.41 (1.30,1.53) |
| Metformin (A10BA02) | Diarrhea | Antidiarrheal (A07) | 193 [116/77] | 1.51 (1.13,2.01) | 1.40 (1.05,1.87) |
| Tricyclic antidepressant (N06AA) | Constipation | Laxative (A06A) | 1469 [851/618] | 1.38 (1.24,1.53) | 1.38 (1.25,1.54) |
| Dihydropyridines (C08CA) | Depression | Antidepressants (N06A) | 1322 [747/575] | 1.30 (1.17,1.45) | 1.34 (1.20,1.50) |
| Dihydropyridines (C08CA) | Edema peripheral | High-ceiling diuretics (C03C) | 1977 [1118/859] | 1.30 (1.19,1.42) | 1.31 (1.20,1.44) |
| Proton pump inhibitors (A02BC) | Bone loss | Bisphosphonates (M05BA) | 499 [284/215] | 1.32 (1.11,1.58) | 1.31 (1.09,1.56) |
| HMG CoA reductase inhibitors (C10AA) (statins) | Sleeplessness | Hypnotics and sedatives (N05C) | 927 [523/404] | 1.29 (1.14,1.47) | 1.30 (1.14,1.48) |
| Selective CCB (cardiac effects) (C08D C09BB10) | Edema peripheral | High-ceiling diuretics (C03C) | 462 [256/206] | 1.24 (1.03,1.49) | 1.29 (1.08,1.56) |
| Corticosteroids (systemic use) (H02A) | Insomnia | Benzodiazepine derivatives (N05BA N05CD) | 3579 [1982/1597] | 1.24 (1.16,1.33) | 1.27 (1.19,1.36) |
| Direct anticoagulants (DOC) (B01AE07 B01AF01 B01AF02) | Depression | Antidepressants (N06A) | 1571 [864/707] | 1.22 (1.11,1.35) | 1.24 (1.13,1.37) |
| Digoxin (C01AA05) | Nausea/dizziness | Antiemetic (A03FA03 N05AD08 A03FA01 A04AD A04AA) | 1244 [680/564] | 1.21 (1.08,1.35) | 1.21 (1.08,1.35) |
| ACE-inhibitors (C09A) | Depression | Antidepressants (N06A) | 1409 [747/662] | 1.13 (1.02,1.25) | 1.13 (1.02,1.26) |

| **Appendix 10:** Summary of statistically significant prescribing cascades occurring before entry to LTCF stratified by those with and without dementia. | | |
| --- | --- | --- |
| **Index medication (ATC)** | **Potential ADR** | **Marker medication (ATC)** |
| **Cascades present in both strata (n = 24)** | | |
| Anticholinergic (R03BB) | Urinary retention in BPH | Dutasteride, Finasteride, Tamsulosin, Dutasteride/Tamsulosin, Alfuzosin, Silodosin (G04CB02, G04CB01, G04CA02, G04CA52, G04CA01, G04CA04) |
| Antihypertensives (C02 C03 C07 C08 C09) | Nausea/dizziness | Antiemetic (A03FA03 N05AD08 A03FA01 A04AD A04AA) |
| Non-steroidal anti-inflammatory drugs (NSAIDs) (M01A) | Heart failure | HF beta-blocker (C07AB07 C07AG02 C07AB02 C07AB12) |
| Non-steroidal anti-inflammatory drugs (NSAIDs) (M01A) | Edema peripheral | High-ceiling diuretics (C03C) |
| Non-steroidal anti-inflammatory drugs (NSAIDs) (M01A) | GI symptoms | Anti-ulcer (A02B) |
| Non-steroidal anti-inflammatory drugs (NSAIDs) (M01A) | Nausea/dizziness | Antiemetic (A03FA03 N05AD08 A03FA01 A04AD A04AA) |
| Benzodiazepine derivatives (N05BA N05CD) | Paradoxical agitation | Antipsychotics (N05A) |
| Beta blocking agents (C07) | Depression | TCA SSRI Other Antidepressant (N06AA N06AB N06AX) |
| Calcium channel blockers (CCB) (C08) | Constipation | Laxative (A06A) |
| Cholinesterase inhibitor (N06DA) | Nausea/dizziness | Antiemetic (A03FA03 N05AD08 A03FA01 A04AD A04AA) |
| Corticosteroids (systemic use) (H02A) | Psychiatric disorders | Antipsychotics (N05A) |
| Corticosteroids (systemic use) (H02A) | Insomnia | Benzodiazepine derivatives (N05BA N05CD) |
| Dihydropyridines (C08CA) | Edema peripheral | High-ceiling diuretics (C03C) |
| Direct anticoagulants (DOC) (B01AE07 B01AF01 B01AF02) | Depression | Antidepressants (N06A) |
| Dopaminergic agent (N04B) | Paradoxical agitation | Antipsychotics (N05A) |
| HMG CoA reductase inhibitors (C10AA) (statins) | Depression | Antidepressants (N06A) |
| HMG CoA reductase inhibitors (C10AA) (statins) | Confusion state | Antipsychotics (N05A) |
| HMG CoA reductase inhibitors (C10AA) (statins) | Sleeplessness | Hypnotics and sedatives (N05C) |
| HMG CoA reductase inhibitors (C10AA) (statins) | Arrhythmia | Antithrombotic (B01AA B01AE B01AF) |
| Metformin (A10BA02) | Diarrhea | Antidiarrheal (A07) |
| Opioids (N02A) | Depression | Antidepressants (N06A) |
| Opioids (N02A) | Nausea/dizziness | Antiemetic (A03FA03 N05AD08 A03FA01 A04AD A04AA) |
| Prazosin (C02CA01) | Urinary incontinence | Oxybutynin (G04BD04) |
| Tricyclic antidepressant (N06AA) | Constipation | Laxative (A06A) |
| **Cascades only among people with dementia (n = 6)** | | |
| Antidepressants (N06A) | Parkinsonism | Anti-Parkinson drugs (N04) |
| Cholinesterase inhibitor (N06DA) | Insomnia | Benzodiazepine derivatives (N05BA N05CD) |
| HMG CoA reductase inhibitors (C10AA) (statins) | Cognitive impairment | Anti-dementia (N06D) |
| Prazosin (C02CA01) | Orthostatic hypotension | Prochlorperazine (A04AD) |
| SSRI/SNRI (N06AX23 N06AX16 N06AX21 N06AB) | Insomnia | Antidepressant (sedating) (N06AX11 N06AX05 N06AA09 N06AA12 N06AA10 N06AX22) |
| Tricyclic antidepressant (N06AA) | Cognitive impairment | Anti-dementia (N06D) |
| **Cascades only among people without dementia (n = 11)** | | |
| ACE-inhibitors (C09A) | Depression | Antidepressants (N06A) |
| Amiodarone (C01BD01) | Hypothyroidism | Levothyroxine (H03AA01) |
| Antiadrenergic/Antimigraine (C02A N02CX) | Depression | TCA SSRI Other Antidepressant (N06AA N06AB N06AX) |
| Digoxin (C01AA05) | Nausea/dizziness | Antiemetic (A03FA03 N05AD08 A03FA01 A04AD A04AA) |
| Dihydropyridines (C08CA) | Depression | Antidepressants (N06A) |
| Erythromycin (J01FA01) | Arrhythmia | Antithrombotic (B01AA B01AE B01AF) |
| Erythromycin (J01FA01) | Arrhythmia | Antiarrhythmics (C01B) |
| HMG CoA reductase inhibitors (C10AA) (statins) | Arrhythmia | Antiarrhythmics (C01B) |
| Proton pump inhibitors (A02BC) | Bone loss | Bisphosphonates (M05BA) |
| Proton pump inhibitors (A02BC) | Clostridium difficile infection | Intestinal anti-infectives (A07A) |
| Selective CCB (cardiac effects) (C08D C09BB10) | Edema peripheral | High-ceiling diuretics (C03C) |

| **Appendix 11:** Statistically significant prescribing cascades during the 18 months after entering LTCF, stratified by dementia status (ordered by adjusted sequence ratio). | | | | | |
| --- | --- | --- | --- | --- | --- |
| **Index Medication** | **Potential ADR** | **Marker Medication** | **Incident users**  **[I-M/M-I]** | **Crude SR**  **(95%CI)** | **Adjusted SR (95%CI)** |
| **Among those living with dementia (n = 40 cascades)** | | | | | |
| Cholinesterase inhibitor (N06DA) | Orthostatic hypotension | Prochlorperazine (A04AD) | 76 [57/19] | 3.00 (1.78,5.04) | 4.03 (2.40,6.78) |
| HMG CoA reductase inhibitors (C10AA) (statins) | Skin soft tissue infection | Antibiotic (J01) | 1937 [1504/433] | 3.47 (3.12,3.87) | 3.34 (3.00,3.72) |
| Cholinesterase inhibitor (N06DA) | Nausea/dizziness | Antiemetic (A03FA03 N05AD08 A03FA01 A04AD A04AA) | 558 [426/132] | 3.23 (2.65,3.92) | 3.22 (2.65,3.91) |
| Digoxin (C01AA05) | Nausea/dizziness | Antiemetic (A03FA03 N05AD08 A03FA01 A04AD A04AA) | 743 [558/185] | 3.02 (2.55,3.56) | 3.07 (2.60,3.62) |
| Antihypertensives (C02 C03 C07 C08 C09) | Nausea/dizziness | Antiemetic (A03FA03 N05AD08 A03FA01 A04AD A04AA) | 2283 [1687/596] | 2.83 (2.58,3.11) | 2.83 (2.58,3.11) |
| HMG CoA reductase inhibitors (C10AA) (statins) | Myalgia/myositis | Non-steroidal anti-inflammatory drugs (NSAIDs) (M01A) | 254 [187/67] | 2.79 (2.11,3.69) | 2.55 (1.93,3.37) |
| HMG CoA reductase inhibitors (C10AA) (statins) | Confusion state | Antipsychotics (N05A) | 895 [630/265] | 2.38 (2.06,2.74) | 2.39 (2.07,2.76) |
| Proton pump inhibitors (A02BC) | Clostridium difficile infection | Intestinal anti-infectives (A07A) | 31 [20/11] | 1.82 (0.87,3.79) | 2.38 (1.14,4.96) |
| HMG CoA reductase inhibitors (C10AA) (statins) | Sleeplessness | Hypnotics and sedatives (N05C) | 494 [341/153] | 2.23 (1.84,2.70) | 2.30 (1.90,2.78) |
| HMG CoA reductase inhibitors (C10AA) (statins) | Depression | Antidepressants (N06A) | 676 [476/200] | 2.38 (2.02,2.81) | 2.27 (1.92,2.67) |
| Dihydropyridines (C08CA) | Edema peripheral | High-ceiling diuretics (C03C) | 782 [544/238] | 2.29 (1.96,2.66) | 2.19 (1.88,2.55) |
| Cholinesterase inhibitor (N06DA) | Diarrhea | Antidiarrheal (A07) | 55 [36/19] | 1.89 (1.09,3.30) | 2.08 (1.19,3.63) |
| Beta blocking agents (C07) | Depression | TCA SSRI Other Antidepressant (N06AA N06AB N06AX) | 1047 [715/332] | 2.15 (1.89,2.45) | 2.02 (1.78,2.31) |
| ACE-inhibitors (C09A) | Cough | Antibacterials (cough) (J01A J01B J01F J01G J01R) | 996 [668/328] | 2.04 (1.78,2.32) | 1.98 (1.74,2.26) |
| HMG CoA reductase inhibitors (C10AA) (statins) | Arrhythmia | Antithrombotic (B01AA B01AE B01AF) | 193 [124/69] | 1.80 (1.34,2.41) | 1.92 (1.43,2.58) |
| ACE-inhibitors (C09A) | Arthritis | Non-steroidal anti-inflammatory drugs (NSAIDs) (M01A) | 303 [191/112] | 1.71 (1.35,2.15) | 1.74 (1.38,2.20) |
| Selective CCB (cardiac effects) (C08D C09BB10) | Depression | TCA SSRI Other Antidepressant (N06AA N06AB N06AX) | 67 [42/25] | 1.68 (1.02,2.76) | 1.72 (1.05,2.83) |
| Opioids (N02A) | Nausea/dizziness | Antiemetic (A03FA03 N05AD08 A03FA01 A04AD A04AA) | 8829 [5509/3320] | 1.66 (1.59,1.73) | 1.66 (1.59,1.74) |
| Selective CCB (cardiac effects) (C08D C09BB10) | Edema peripheral | High-ceiling diuretics (C03C) | 83 [51/32] | 1.59 (1.02,2.48) | 1.66 (1.07,2.58) |
| Antihypertensives (C02 C03 C07 C08 C09) | Nausea/dizziness | Prochlorperazine (A04AD) | 260 [163/97] | 1.68 (1.31,2.16) | 1.65 (1.29,2.13) |
| Laxative (A06A) | Diarrhea | Antidiarrheal (A07) | 666 [417/249] | 1.67 (1.43,1.96) | 1.58 (1.35,1.84) |
| Latanoprost (S01EE01) | Edema peripheral | High-ceiling diuretics (C03C) | 154 [92/62] | 1.48 (1.08,2.05) | 1.57 (1.14,2.17) |
| ACE-inhibitors (C09A) | Depression | Antidepressants (N06A) | 797 [485/312] | 1.55 (1.35,1.79) | 1.53 (1.33,1.77) |
| Non-steroidal anti-inflammatory drugs (NSAIDs) (M01A) | Nausea/dizziness | Antiemetic (A03FA03 N05AD08 A03FA01 A04AD A04AA) | 1488 [890/598] | 1.49 (1.34,1.65) | 1.50 (1.36,1.67) |
| Dihydropyridines (C08CA) | Depression | Antidepressants (N06A) | 701 [419/282] | 1.49 (1.28,1.73) | 1.45 (1.24,1.68) |
| Benzodiazepine derivatives (N05BA N05CD) | Nausea/dizziness | Prochlorperazine (A04AD) | 585 [341/244] | 1.40 (1.19,1.65) | 1.44 (1.22,1.70) |
| Antipsychotics (N05A) | Parkinsonism/Extrapyramidal symptoms | Anti-Parkinson drugs (N04) | 408 [237/171] | 1.39 (1.14,1.69) | 1.43 (1.17,1.74) |
| Antidepressants (N06A) | Parkinsonism | Anti-Parkinson drugs (N04) | 288 [161/127] | 1.27 (1.00,1.60) | 1.41 (1.11,1.77) |
| Cholinesterase inhibitor (N06DA) | Peptic ulcer | Proton pump inhibitors (A02BC) | 270 [152/118] | 1.29 (1.01,1.64) | 1.39 (1.10,1.77) |
| Antiepileptics (N03A) | Nausea/dizziness | Antiemetic (A03FA03 N05AD08 A03FA01 A04AD A04AA) | 1434 [828/606] | 1.37 (1.23,1.52) | 1.38 (1.24,1.53) |
| Proton pump inhibitors (A02BC) | Pneumonia | Antibiotic (J01) | 4571 [2652/1919] | 1.38 (1.30,1.47) | 1.37 (1.29,1.46) |
| Benzodiazepine derivatives (N05BA N05CD) | Paradoxical agitation | Antipsychotics (N05A) | 7688 [4442/3246] | 1.37 (1.31,1.43) | 1.36 (1.30,1.43) |
| Antiepileptics (other) (N03AX) | Urinary tract infection (UTI) | Antibacterials (UTI) (J01C J01D J01E J01M J01X) | 538 [314/224] | 1.40 (1.18,1.66) | 1.36 (1.15,1.61) |
| Direct anticoagulants (DOC) (B01AE07 B01AF01 B01AF02) | Depression | Antidepressants (N06A) | 587 [336/251] | 1.34 (1.14,1.58) | 1.35 (1.14,1.59) |
| Calcium channel blockers (CCB) (C08) | Constipation | Laxative (A06A) | 1400 [803/597] | 1.35 (1.21,1.50) | 1.34 (1.21,1.49) |
| Benzodiazepine derivatives (N05BA N05CD) | Edema peripheral | High-ceiling diuretics (C03C) | 4147 [2364/1783] | 1.33 (1.25,1.41) | 1.32 (1.24,1.40) |
| SSRI/SNRI (N06AX23 N06AX16 N06AX21 N06AB) | Insomnia | Antidepressant (sedating) (N06AX11 N06AX05 N06AA09 N06AA12 N06AA10 N06AX22) | 1437 [801/636] | 1.26 (1.13,1.40) | 1.28 (1.16,1.42) |
| ACE-inhibitors (C09A) | Cough | Adrenergics/inhalants (R03A) | 635 [358/277] | 1.29 (1.10,1.51) | 1.26 (1.07,1.47) |
| Antiepileptics (N03A) | Rash topical | Corticosteroid (D07) | 1202 [674/528] | 1.28 (1.14,1.43) | 1.22 (1.09,1.37) |
| Proton pump inhibitors (A02BC) | Bone loss | Denosumab (M05BX04) | 846 [472/374] | 1.26 (1.10,1.45) | 1.22 (1.07,1.40) |
| **Among those not living with dementia (n = 35 cascades)** | | | | | |
| HMG CoA reductase inhibitors (C10AA) (statins) | Confusion state | Antipsychotics (N05A) | 488 [402/86] | 4.67 (3.70,5.90) | 4.61 (3.66,5.82) |
| Proton pump inhibitors (A02BC) | Clostridium difficile infection | Intestinal anti-infectives (A07A) | 58 [44/14] | 3.14 (1.72,5.73) | 3.61 (1.98,6.58) |
| HMG CoA reductase inhibitors (C10AA) (statins) | Skin soft tissue infection | Antibiotic (J01) | 2381 [1816/565] | 3.21 (2.92,3.53) | 3.13 (2.85,3.44) |
| HMG CoA reductase inhibitors (C10AA) (statins) | Arrhythmia | Antiarrhythmics (C01B) | 42 [30/12] | 2.50 (1.28,4.88) | 3.13 (1.60,6.10) |
| Metformin (A10BA02) | Diarrhea | Antidiarrheal (A07) | 100 [73/27] | 2.70 (1.74,4.20) | 2.94 (1.89,4.57) |
| Digoxin (C01AA05) | Nausea/dizziness | Antiemetic (A03FA03 N05AD08 A03FA01 A04AD A04AA) | 1307 [955/352] | 2.71 (2.40,3.07) | 2.73 (2.42,3.09) |
| Antihypertensives (C02 C03 C07 C08 C09) | Nausea/dizziness | Antiemetic (A03FA03 N05AD08 A03FA01 A04AD A04AA) | 2680 [1941/739] | 2.63 (2.41,2.86) | 2.56 (2.35,2.79) |
| HMG CoA reductase inhibitors (C10AA) (statins) | Myalgia/myositis | Non-steroidal anti-inflammatory drugs (NSAIDs) (M01A) | 327 [239/88] | 2.72 (2.13,3.47) | 2.51 (1.96,3.20) |
| ACE-inhibitors (C09A) | Cough | Antitussive (R05F R05D) | 44 [29/15] | 1.93 (1.04,3.61) | 2.46 (1.32,4.59) |
| Amiodarone (C01BD01) | Hypothyroidism | Levothyroxine (H03AA01) | 65 [39/26] | 1.50 (0.91,2.46) | 2.35 (1.43,3.86) |
| HMG CoA reductase inhibitors (C10AA) (statins) | Arrhythmia | Antithrombotic (B01AA B01AE B01AF) | 342 [233/109] | 2.14 (1.70,2.68) | 2.28 (1.81,2.86) |
| HMG CoA reductase inhibitors (C10AA) (statins) | Depression | Antidepressants (N06A) | 757 [525/232] | 2.26 (1.94,2.64) | 2.20 (1.89,2.57) |
| Benzodiazepine derivatives (N05BA N05CD) | Paradoxical agitation | Antipsychotics (N05A) | 3036 [2048/988] | 2.07 (1.92,2.24) | 2.06 (1.91,2.23) |
| HMG CoA reductase inhibitors (C10AA) (statins) | Sleeplessness | Hypnotics and sedatives (N05C) | 670 [448/222] | 2.02 (1.72,2.37) | 2.05 (1.74,2.41) |
| Dopaminergic agent (N04B) | Paradoxical agitation | Antipsychotics (N05A) | 217 [138/79] | 1.75 (1.32,2.30) | 2.00 (1.51,2.63) |
| Benzodiazepine derivatives (N05BA N05CD) | Cognitive impairment | Anti-dementia (N06D) | 237 [159/78] | 2.04 (1.55,2.67) | 1.89 (1.45,2.48) |
| Selective CCB (cardiac effects) (C08D C09BB10) | Depression | TCA SSRI Other Antidepressant (N06AA N06AB N06AX) | 96 [62/34] | 1.82 (1.20,2.77) | 1.84 (1.21,2.80) |
| Antihypertensives (C02 C03 C07 C08 C09) | Nausea/dizziness | Prochlorperazine (A04AD) | 460 [300/160] | 1.88 (1.55,2.27) | 1.83 (1.51,2.21) |
| Dihydropyridines (C08CA) | Edema peripheral | High-ceiling diuretics (C03C) | 1163 [754/409] | 1.84 (1.63,2.08) | 1.82 (1.61,2.05) |
| Corticosteroids (systemic use) (H02A) | Psychiatric disorders | Antipsychotics (N05A) | 1623 [1023/600] | 1.71 (1.54,1.89) | 1.75 (1.58,1.94) |
| Beta blocking agents (C07) | Depression | TCA SSRI Other Antidepressant (N06AA N06AB N06AX) | 1296 [819/477] | 1.72 (1.53,1.92) | 1.69 (1.51,1.89) |
| Opioids (N02A) | Nausea/dizziness | Antiemetic (A03FA03 N05AD08 A03FA01 A04AD A04AA) | 10115 [6307/3808] | 1.66 (1.59,1.72) | 1.65 (1.58,1.72) |
| ACE-inhibitors (C09A) | Cough | Antibacterials (cough) (J01A J01B J01F J01G J01R) | 1577 [974/603] | 1.62 (1.46,1.79) | 1.58 (1.43,1.75) |
| Proton pump inhibitors (A02BC) | Pneumonia | Antibiotic (J01) | 5532 [3331/2201] | 1.51 (1.43,1.60) | 1.49 (1.41,1.57) |
| Antiepileptics (other) (N03AX) | Urinary tract infection (UTI) | Antibacterials (UTI) (J01C J01D J01E J01M J01X) | 764 [458/306] | 1.50 (1.30,1.73) | 1.48 (1.28,1.71) |
| ACE-inhibitors (C09A) | Arthritis | Non-steroidal anti-inflammatory drugs (NSAIDs) (M01A) | 392 [226/166] | 1.36 (1.11,1.66) | 1.40 (1.14,1.71) |
| Dihydropyridines (C08CA) | Depression | Antidepressants (N06A) | 850 [487/363] | 1.34 (1.17,1.54) | 1.37 (1.20,1.57) |
| Benzodiazepine derivatives (N05BA N05CD) | Nausea/dizziness | Prochlorperazine (A04AD) | 1004 [581/423] | 1.37 (1.21,1.56) | 1.34 (1.18,1.52) |
| Proton pump inhibitors (A02BC) | Bone loss | Denosumab (M05BX04) | 1043 [605/438] | 1.38 (1.22,1.56) | 1.34 (1.18,1.51) |
| ACE-inhibitors (C09A) | Depression | Antidepressants (N06A) | 925 [526/399] | 1.32 (1.16,1.50) | 1.30 (1.14,1.48) |
| High-ceiling diuretics (C03C) | Diuresis excessive | Benign prostatic hypertrophy (G04C) | 368 [207/161] | 1.29 (1.05,1.58) | 1.29 (1.05,1.59) |
| Laxative (A06A) | Diarrhea | Antidiarrheal (A07) | 938 [531/407] | 1.30 (1.15,1.48) | 1.28 (1.12,1.45) |
| Antiepileptics (N03A) | Rash topical | Corticosteroid (D07) | 895 [488/407] | 1.20 (1.05,1.37) | 1.21 (1.06,1.38) |
| Direct anticoagulants (DOC) (B01AE07 B01AF01 B01AF02) | Depression | Antidepressants (N06A) | 937 [502/435] | 1.15 (1.01,1.31) | 1.18 (1.04,1.35) |
| Non-steroidal anti-inflammatory drugs (NSAIDs) (M01A) | Nausea/dizziness | Antiemetic (A03FA03 N05AD08 A03FA01 A04AD A04AA) | 2012 [1060/952] | 1.11 (1.02,1.22) | 1.12 (1.03,1.22) |

| **Appendix 12:** Summary of statistically significant prescribing cascades occurring after entry to LTCF stratified by those with and without dementia. | | |
| --- | --- | --- |
| **Index medication (ATC)** | **Potential ADR** | **Marker medication (ATC)** |
| **Cascades present in both strata (n = 27)** | | |
| ACE-inhibitors (C09A) | Cough | Antibacterials (cough) (J01A J01B J01F J01G J01R) |
| ACE-inhibitors (C09A) | Arthritis | Non-steroidal anti-inflammatory drugs (NSAIDs) (M01A) |
| ACE-inhibitors (C09A) | Depression | Antidepressants (N06A) |
| Antiepileptics (N03A) | Rash topical | Corticosteroid (D07) |
| Antiepileptics (other) (N03AX) | Urinary tract infection (UTI) | Antibacterials (UTI) (J01C J01D J01E J01M J01X) |
| Antihypertensives (C02 C03 C07 C08 C09) | Nausea/dizziness | Antiemetic (A03FA03 N05AD08 A03FA01 A04AD A04AA) |
| Antihypertensives (C02 C03 C07 C08 C09) | Nausea/dizziness | Prochlorperazine (A04AD) |
| Non-steroidal anti-inflammatory drugs (NSAIDs) (M01A) | Nausea/dizziness | Antiemetic (A03FA03 N05AD08 A03FA01 A04AD A04AA) |
| Benzodiazepine derivatives (N05BA N05CD) | Paradoxical agitation | Antipsychotics (N05A) |
| Benzodiazepine derivatives (N05BA N05CD) | Nausea/dizziness | Prochlorperazine (A04AD) |
| Beta blocking agents (C07) | Depression | TCA SSRI Other Antidepressant (N06AA N06AB N06AX) |
| Digoxin (C01AA05) | Nausea/dizziness | Antiemetic (A03FA03 N05AD08 A03FA01 A04AD A04AA) |
| Dihydropyridines (C08CA) | Edema peripheral | High-ceiling diuretics (C03C) |
| Dihydropyridines (C08CA) | Depression | Antidepressants (N06A) |
| Direct anticoagulants (DOC) (B01AE07 B01AF01 B01AF02) | Depression | Antidepressants (N06A) |
| HMG CoA reductase inhibitors (C10AA) (statins) | Skin soft tissue infection | Antibiotic (J01) |
| HMG CoA reductase inhibitors (C10AA) (statins) | Myalgia/myositis | Non-steroidal anti-inflammatory drugs (NSAIDs) (M01A) |
| HMG CoA reductase inhibitors (C10AA) (statins) | Confusion state | Antipsychotics (N05A) |
| HMG CoA reductase inhibitors (C10AA) (statins) | Sleeplessness | Hypnotics and sedatives (N05C) |
| HMG CoA reductase inhibitors (C10AA) (statins) | Depression | Antidepressants (N06A) |
| HMG CoA reductase inhibitors (C10AA) (statins) | Arrhythmia | Antithrombotic (B01AA B01AE B01AF) |
| Laxative (A06A) | Diarrhea | Antidiarrheal (A07) |
| Opioids (N02A) | Nausea/dizziness | Antiemetic (A03FA03 N05AD08 A03FA01 A04AD A04AA) |
| Proton pump inhibitors (A02BC) | Clostridium difficile infection | Intestinal anti-infectives (A07A) |
| Proton pump inhibitors (A02BC) | Bone loss | Denosumab (M05BX04) |
| Proton pump inhibitors (A02BC) | Pneumonia | Antibiotic (J01) |
| Selective CCB (cardiac effects) (C08D C09BB10) | Depression | TCA SSRI Other Antidepressant (N06AA N06AB N06AX) |
| **Cascades only among people with dementia (n = 13)** | | |
| ACE-inhibitors (C09A) | Cough | Adrenergics/inhalants (R03A) |
| Antidepressants (N06A) | Parkinsonism | Anti-Parkinson drugs (N04) |
| Antiepileptics (N03A) | Nausea/dizziness | Antiemetic (A03FA03 N05AD08 A03FA01 A04AD A04AA) |
| Antipsychotics (N05A) | Parkinsonism/Extrapyramidal symptoms | Anti-Parkinson drugs (N04) |
| Benzodiazepine derivatives (N05BA N05CD) | Edema peripheral | High-ceiling diuretics (C03C) |
| Calcium channel blockers (CCB) (C08) | Constipation | Laxative (A06A) |
| Cholinesterase inhibitor (N06DA) | Orthostatic hypotension | Prochlorperazine (A04AD) |
| Cholinesterase inhibitor (N06DA) | Peptic ulcer | Proton pump inhibitors (A02BC) |
| Cholinesterase inhibitor (N06DA) | Diarrhea | Antidiarrheal (A07) |
| Cholinesterase inhibitor (N06DA) | Nausea/dizziness | Antiemetic (A03FA03 N05AD08 A03FA01 A04AD A04AA) |
| Latanoprost (S01EE01) | Edema peripheral | High-ceiling diuretics (C03C) |
| Selective CCB (cardiac effects) (C08D C09BB10) | Edema peripheral | High-ceiling diuretics (C03C) |
| SSRI/SNRI (N06AX23 N06AX16 N06AX21 N06AB) | Insomnia | Antidepressant (sedating) (N06AX11 N06AX05 N06AA09 N06AA12 N06AA10 N06AX22) |
| **Cascades only among people without dementia (n = 8)** | | |
| ACE-inhibitors (C09A) | Cough | Antitussive (R05F R05D) |
| Amiodarone (C01BD01) | Hypothyroidism | Levothyroxine (H03AA01) |
| Benzodiazepine derivatives (N05BA N05CD) | Cognitive impairment | Anti-dementia (N06D) |
| Corticosteroids (systemic use) (H02A) | Psychiatric disorders | Antipsychotics (N05A) |
| Dopaminergic agent (N04B) | Paradoxical agitation | Antipsychotics (N05A) |
| High-ceiling diuretics (C03C) | Diuresis excessive | Benign prostatic hypertrophy (G04C) |
| HMG CoA reductase inhibitors (C10AA) (statins) | Arrhythmia | Antiarrhythmics (C01B) |
| Metformin (A10BA02) | Diarrhea | Antidiarrheal (A07) |
